# Plasmodesmata display dynamic local and systemic redox responses during plant stress

**DOI:** 10.1093/plcell/koag192

**Published:** 2026-06-22

**Authors:** Niraj Kumar Vishwakarma, Md Abdur Razzak, Vishnu Mishra, Timothy Chaya, Jeffrey L Caplan, Jung-Youn Lee

**Affiliations:** Department of Plant and Soil Sciences, University of Delaware, Newark, DE 19713, United States; Delaware Biotechnology Institute, University of Delaware, Newark, DE 19713, United States; Department of Plant and Soil Sciences, University of Delaware, Newark, DE 19713, United States; Delaware Biotechnology Institute, University of Delaware, Newark, DE 19713, United States; Department of Plant and Soil Sciences, University of Delaware, Newark, DE 19713, United States; Delaware Biotechnology Institute, University of Delaware, Newark, DE 19713, United States; Department of Plant and Soil Sciences, University of Delaware, Newark, DE 19713, United States; Delaware Biotechnology Institute, University of Delaware, Newark, DE 19713, United States; Department of Plant and Soil Sciences, University of Delaware, Newark, DE 19713, United States; Delaware Biotechnology Institute, University of Delaware, Newark, DE 19713, United States; Department of Biological Sciences, University of Delaware, Newark, DE 19716, United States; Department of Plant and Soil Sciences, University of Delaware, Newark, DE 19713, United States; Delaware Biotechnology Institute, University of Delaware, Newark, DE 19713, United States; Department of Biological Sciences, University of Delaware, Newark, DE 19716, United States

## Abstract

Hydrogen peroxide (H_2_O_2_) is a potent reactive oxygen species that plays a crucial role as a versatile signaling molecule for cellular function and vitality. Recent experimental evidence indicates that H_2_O_2_ affects cell-to-cell communication through plasmodesmata, tiny cytoplasmic nanopores connecting adjacent plant cells. H_2_O_2_-dependent systemic signaling has also been reported to involve plasmodesmal function in some contexts, although the dominant routes and messengers underlying rapid long-distance signaling remain under active debate. Nevertheless, direct monitoring of redox dynamics at plasmodesmata in live tissues has remained challenging. In this study, we developed a plasmodesmata-localized HyPer7 (Pd-HyPer7) reporter to investigate H_2_O_2_ dynamics at plasmodesmata in response to exogenous redox stressors and plant stresses, including cold and mechanical wounding. Pd-HyPer7 showed response characteristics that differed from the HyPer7 reporters localized to the cytosol, plasma membrane, and chloroplasts under the conditions tested, indicating that redox responses at plasmodesmata are distinguishable from these compartments. Notably, during mechanical wounding, both the cytosol and plasmodesmata showed transient redox responses with broadly similar temporal profiles in local tissues. In systemic tissues, however, the responses were temporally separated, with plasmodesmal oxidation peaking well after the cytosolic response. This timing relationship is consistent with plasmodesmata acting downstream of early systemic wound signaling, rather than simply mirroring cytosolic redox dynamics. Together, our results establish Pd-HyPer7 as a tool for monitoring plasmodesmal redox dynamics and support a model in which plasmodesmata participate in spatially and temporally regulated redox responses during plant stress.

## Introduction

A well-orchestrated signal perception, processing, and relay across cellular boundaries is vital to multicellular organisms to grow, develop healthily, and ensure survival and reproduction. This aspect is especially critical for plants given their cellular immobility and sessile lifestyle. As an adaptation, plants have evolved a unique signal relay system through direct intercellular bridges called plasmodesmata. Plasmodesmata are tiny pores that are formed between adjacent cells to facilitate the transport of various types of small diffusible molecules, such as nutrients, ions, and hormones, as well as large mobile molecules including RNAs and proteins. It is now well established that plasmodesmata are highly dynamic channels undergoing rapid changes in their permeability in response to both internal signals and external challenges, as discussed in recent reviews (Sager and Lee 2014; Bayer and Benitez-Alfonso 2024; Tee and Faulkner 2024; Zanini and Burch-Smith 2024). Accumulating evidence also indicates that the dynamic responses at the plasmodesmata are enabled by regulatory mechanisms that integrate plasmodesmata with cellular signaling cascades to bring about coordinated responses across cellular boundaries, not only locally among adjacent cells, but also systemically throughout the whole plant body (Lee et al. 2011; Wang et al. 2011, 2023; Faulkner et al. 2013; Cui and Lee 2016; Lim et al. 2016; Sager et al. 2020; Tee et al. 2023; Li et al. 2024). Given their central role in plant-wide communication, it is not surprising that perturbation of plasmodesmal regulation results in loss of adaptive responses.

Among the various cellular signaling molecules, hydrogen peroxide (H_2_O_2_) emerged as a key molecule crucial for establishing systemic acclimation to abiotic stress in plants (Petrov and Van Breusegem 2012; Mignolet-Spruyt et al. 2016; Sies 2017; Waszczak et al. 2018). H_2_O_2_, a potent reactive oxygen species (ROS), is required to regulate cellular processes that are essential for maintaining redox homeostasis and survival in both animals and plants. In animal cells, H_2_O_2_ accumulation is triggered during cell proliferation, differentiation, or programmed cell death in response to specific growth factors, cytokines, or oxidative stressors (Veal and Day 2011). Similarly, in plants, exposure to environmental stressors such as high light, extreme temperatures, mechanical wounding, or pathogen attack elicits an increase in H_2_O_2_ levels (Mittler et al. 2011; Smirnoff and Arnaud 2019). This then triggers a cascade of signaling events that facilitate plant adaptation to stress conditions and maintenance of redox homeostasis. In plants, the production of H_2_O_2_ can be induced rapidly in varying amounts and durations by an array of environmental stimuli and developmental cues (Waszczak et al. 2018). While the burst or increase in H_2_O_2_ levels occurs both extracellularly in the apoplast and intracellularly in various organelles, including chloroplasts, mitochondria, and peroxisomes, excessive H_2_O_2_ accumulation can be detrimental to cell vitality (Mignolet-Spruyt et al. 2016; Podgorska et al. 2017; Smirnoff and Arnaud 2019). Thus, cells maintain precise spatiotemporal redox homeostasis by tightly controlling H_2_O_2_ levels via a network of antioxidant enzymes localized not only within each compartment, but also in the cytosol (Foyer and Noctor 2011; Foyer and Kunert 2024).

As a signaling molecule, H_2_O_2_ also plays a crucial role in modulating cell-cell communication in both plant and animal cells (Fichman et al. 2023). In animal cells, H_2_O_2_ can affect neighboring cells through localized production and controlled diffusion, often facilitated by specific membrane channels (Miller et al. 2010; Ledo et al. 2022). Interestingly, in animal cells, H_2_O_2_ can stimulate the formation of membrane-lined, direct cell-cell communication channels analogous to plasmodesmata (Wang et al. 2011; Gousset et al. 2013; Rustom 2016; Liu et al. 2018). Whether H_2_O_2_ may have a parallel role as a signal to induce de novo biogenesis of plasmodesmata in plants remains an open question, and it is still unclear if H_2_O_2_ itself moves through plasmodesmata during its propagation. However, one notable observation is that, unlike in animals, H_2_O_2_ signals can rapidly propagate both locally and systemically in plants, and this process appears to require genes that regulate plasmodesmal permeability (Fichman et al. 2021). Indeed, RBOHD-generated ROS have been implicated in regulating callose deposition and immune signaling at plasmodesmata (Cheval et al. 2020; Vu et al. 2022; Tee et al. 2023). Notably, while H_2_O_2_ propagation appears to require functionally intact plasmodesmata, studies have also shown that H_2_O_2_ itself alters plasmodesmal permeability. For instance, using cytochemical staining, a study has shown that lateral meristematic cells accumulate H_2_O_2_ locally at the cell wall close to plasmodesmata (Ehlers and van Bel 2010), while another study demonstrated that chemically manipulating organellar redox states impacts cell-to-cell movement through plasmodesmata (Stonebloom et al. 2012). In a separate investigation, direct exogenous application of H_2_O_2_ has been shown to modulate plasmodesmal permeability between root meristematic cells in *Arabidopsis* seedlings (Rutschow et al. 2011). More recently, we have shown that exogenous H_2_O_2_, as well as endogenously produced H_2_O_2_ induced by mechanical wounding, results in a rapid plasmodesmal closure in leaf tissues of mature *Arabidopsis* plants (Cui and Lee 2016). In that study, we had speculated the possibility that H_2_O_2_ directly targets a specific plasmodesmal regulatory machinery given the rapidity of H_2_O_2_-induced plasmodesmal closure. Nevertheless, the precise molecular mechanism of this regulation remains obscure. In particular, it is unknown whether H_2_O_2_ accumulates directly at the plasmodesmata to alter plasmodesmal permeability or it acts indirectly through signal transduction.

In this study, to address whether H_2_O_2_ accumulates locally at plasmodesmata by monitoring real-time ROS dynamics at these intercellular bridges, we employed the genetically encoded ultrasensitive H_2_O_2_ redox sensor, HyPer7 (Pak et al. 2020) targeted to plasmodesmata. Using this approach, we performed a comparative study by targeting the same HyPer7 sensor to different subcellular compartments, including chloroplasts, cytosol, and plasma membrane. Through this investigation, we succeeded in visualizing how redox states change at plasmodesmata in real-time in response to defined oxidative, reductive, and stress treatments. Together, our findings reveal that plasmodesmata display sensitive and dynamic redox responses during stress and establish Pd-HyPer7 as a tool for investigating redox signaling across cellular interfaces.

## Results

### Generation of an ultrasensitive H_2_O_2_ sensor localized to plasmodesmata

**Figure 1:**
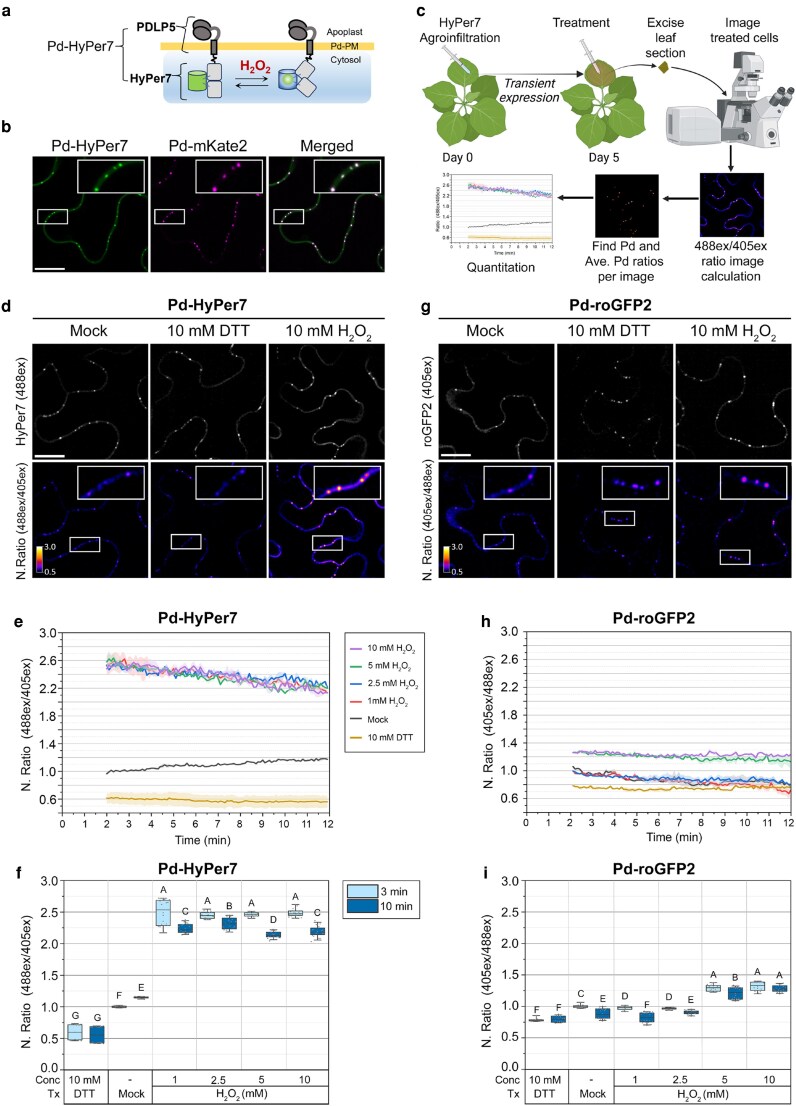
Pd-HyPer7 reports redox dynamics at plasmodesmata. (a) A cartoon illustrating the structure and orientation of the recombinant Pd-HyPer7. HyPer7 is fused to the C-terminus of PDLP5 to report redox dynamics at the cytosolic face of plasmodesmata. (b) A representative confocal image showing punctate fluorescent patterns of Pd-HyPer7 co-localizing with a far-red fluorescent Pd-marker, Pd-mKate2. (c). A schema illustrating the experimental workflow, which involves transient reporter expression in *N. benthamiana,* chemical treatment, ratiometric confocal imaging, and image data processing and quantitative analyses. Created in BioRender. (d) to (f) Time-lapse ratiometric imaging of Pd-HyPer7 under different redox conditions. (d) Representative fluorescence images of Pd-HyPer7. Top panel, 488 nm excitation; and bottom panel, ratio image (488 nm ex./405 nm ex.). Images show responses to mock treatment (water), 10 mM DTT, and 10 mM H_2_O_2_ selected at 3-mpt. Ratio values are represented by pseudocolor “Fire” LUT scaled from 0.5 to 3.0. Scale bar, 20 µm. Same LUT scale and scale bar apply to all ratio images. (e) Time-lapse of Pd-HyPer7 ratios over a 10-minute period following different treatments including varying concentrations of H_2_O_2_. The graph represents data from a total of *n* = 6 images from at least four plants per treatment, combined from two experiments, which were performed independently to ensure reproducibility. Shading represents SE. (f) Box plot showing fluorescence ratios (488 nm ex./405 nm ex.) of Pd-HyPer7 at 3- and 10-mpt selected from data in (e). (g) to (i) Comparative time-lapse ratiometric imaging of Pd-roGFP2. For roGFP2, the plotted ratio is 405ex/488ex. Experiments and data collection were performed as described for Pd-HyPer7. (g) Representative fluorescence images. (h) Time-lapse represents data from a total of *n* = 6 images from at least four plants per treatment, combined from two replicate experiments. Shading represents SE. (i) Box plot showing ratios at 3- and 10-mpt selected from data in (h), where indicated (N. Ratio) ratios were normalized to each sensor's mock at 3-mpt collected in the same experimental session. Each data point represents the mean ratio of plasmodesmata captured within each image, which were autodetected using custom ImageJ macro scripts, created to perform background subtraction, thresholding, and ratio calculation. Box plots (in all figures) show the interquartile range (25th to 75th percentile) with the median line, whiskers to the 5th and 95th percentiles, and individual data points. Statistical analyses were performed across all treatment × time point combinations in a single Kruskal–Wallis test followed by Conover's post-hoc test with Benjamini–Hochberg correction (*α* = 0.05). Letters are assigned with “A” corresponding to the highest group mean; groups sharing the same letter are not significantly different.

To examine the redox status and dynamics at the plasmodesmata, we evaluated the suitability and efficacy of a recently developed H_2_O_2_ sensor named HyPer7 (Pak et al. 2020). HyPer7 has been shown to exhibit superb sensitivity and dynamic range along with pH insensitivity both in mammalian and plant cells (Pak et al. 2020; Ugalde et al. 2021b; Dopp et al. 2023). To localize and orient the sensor on the cytosolic face of plasmodesmata, we fused HyPer7 to the C-terminal end of a well-known plasmodesmal membrane protein, PDLP5 (Lee et al. 2011), with a flexible linker inserted between them (Fig. 1a). PDLP5 is a type-I transmembrane protein that specifically localizes to plasmodesmata and regulates plasmodesmal permeability by stimulating callose deposition, thereby restricting molecular movement between cells (Lee et al. 2011). It plays a critical role in salicylic acid (SA)-mediated innate immunity in the aerial tissues and auxin-dependent lateral root development (Wang et al. 2013; Lim et al. 2016; Sager et al. 2020). In the current study, PDLP5 was chosen to localize HyPer7 to plasmodesmata because of its highly specific plasmodesmal localization in various plants, including *Arabidopsis* and *Nicotiana benthamiana,* and more importantly, it is not involved in mediating H_2_O_2_-dependent regulation of plasmodesmal permeability (Cui and Lee 2016). Typically, plasmodesmata localization is validated with aniline blue staining of plasmodesmal callose, but HyPer7's fluorescence is nearly identical. Thus, we took a two-step approach. First, we fused PDLP5 to mKate2 (Pd-mKate2), a far-red fluorescent protein (Shcherbo et al. 2009) with no spectral overlap with HyPer7, and verified its plasmodesmal localization using aniline blue staining (Figure S1). Then, we separately colocalized the validated Pd-mKate2 with PDLP5-HyPer7 (herein, Pd-HyPer7) to characteristic punctate signals along the cell peripheries typical of plasmodesmata in epidermal cells (Fig. 1b).

For the localization and ratio imaging experiments, the HyPer7 sensor was expressed using *Agrobacterium-*mediated transient expression in 3.5-week-old *N. benthamiana* plants and imaged 5 d later by spinning disk confocal microscopy. Figure 1c shows an overview of the workflow. Small pieces of treated leaves expressing the sensor were excised and immediately mounted on a chamber slide for imaging. HyPer7 has one emission peak at 516 nm, but two excitation peaks. When oxidized by H_2_O_2_, its 500 nm excitation peak increases and its 400 nm excitation peak decreases (Pak et al. 2020). For our experiments, we used fast, sequential imaging of HyPer7 fluorescence with 405 nm excitation (405ex) and 488 nm excitation (488ex) on a spinning disk confocal microscope. The collected raw image data were then batch processed to generate a ratiometric image (488ex/405ex) to find and measure the 488ex/405ex ratio at subcellular locations, and then displayed as ratiometric images using a Fire look-up table (LUT) (Figure S2 and Methods). For the time-lapse experiments, our ratio measurements began at 2 min post-treatment (mpt), reflecting the minimum time required for sample preparation, and continued until 12-mpt.

To evaluate the functionality of Pd-HyPer7 to detect changes in plasmodesmal H_2_O_2_, we infiltrated small regions of leaves with highly oxidative (10 mM H_2_O_2_), highly reductive (10 mM dithiothreitol [DTT]), or mock control (water) treatments. Tissue excisions were mounted in the treatments and imaged using spinning disk confocal microscopy. Compared to the mock control, the 488ex/405ex ratio of Pd-HyPer7 was higher with H_2_O_2_ and lower with DTT treatments (Fig. 1d). As an additional control, we targeted HyPer7 to plasmodesmata using a nonfunctional PDLP5 (mTM). mTM localizes to plasmodesmata, but unlike wild-type (WT) PDLP5, it is impaired in plasmodesmata-regulating function (Wang et al. 2020). The plasmodesmal localization of mTM-HyPer7 was confirmed by localization with Pd-mKate2 in *N. benthamiana* (Figure S3). Importantly, the quantitative analyses of the ratio images revealed that Pd-HyPer7 (WT PDLP5) and mTM-HyPer7 (mutant PDLP5) were both functional at plasmodesmata with no statistical difference in the ratio at 3-mpt under mock conditions (Figure S4). Moreover, upon the H_2_O_2_ treatment, both sensors similarly showed an over 2-fold increase relative to their 3-mpt mock ratio values. These results indicate that either the WT or the mutant form of PDLP5 can be used to target a functional H_2_O_2_ sensor to plasmodesmata. However, for some unknown reasons, we were unable to isolate *Arabidopsis* transgenic lines that express mTM-HyPer7 at sufficiently detectable levels. Therefore, WT Pd-HyPer7 was used in all our subsequent investigations to maintain consistency across *N. benthamiana* and *Arabidopsis* experiments. For *Arabidopsis* studies, we screened for transgenic Pd-HyPer7 lines that had a low expression level that was high enough for microscopic H_2_O_2_ measurements but did not cause an overexpression growth phenotype as discussed in a later Results section.

### HyPer7 is suitable for measuring redox responses at plasmodesmata

Having confirmed the localization and functionality of Pd-HyPer7, we next investigated the redox dynamics of plasmodesmata in detail using the *N. benthamiana* transient expression system as described earlier. Specifically, to systematically characterize these dynamics under different redox conditions, we performed time-lapse ratio imaging using either DTT or varying concentrations of H_2_O_2_ (1 to 10 mM) (Fig. 1, d to f). Additionally, to capture the temporal nature of these redox responses and examine significant changes between the early and late responses within and between treatments, comparisons of the ratios at 3-mpt and 10-mpt were conducted (Fig. 1f). Because excised tissues remained submerged in treatment for the duration of imaging, these traces report dynamics under sustained exposure. In mock-treated plants using water, Pd-HyPer7 exhibited a relatively stable ratio with small increases over 10 min, consistent with the sensor's high sensitivity to small H_2_O_2_ fluctuations during imaging (Fig. 1, e and f). In contrast, ratio relative to mock, DTT treatment decreased the Pd-HyPer7 ratio by 40% and H_2_O_2_ increased it more than 2-fold. Interestingly, this response saturated at 1 mM H_2_O_2_, as higher concentrations produced no additional oxidation, suggesting this as a potential maximum oxidation level (Fig. 1e). Over the 10-minute time-lapse, this initial oxidation gradually decreased, possibly reflecting an activation of local antioxidant systems. Collectively, these results reveal that plasmodesmal redox state is highly dynamic and sensitive, capable of generating rapid responses to both oxidative and reductive stressors.

To further characterize the plasmodesmal redox environment, we examined a second sensor, roGFP2, which responds to changes in the glutathione redox state rather than directly to H_2_O_2_ levels (Morgan et al. 2011). To this end, we produced Pd-roGFP2 using PDLP5 for targeting and confirmed its plasmodesmal localization (Figure S5). The excitation response of roGFP2 is opposite to that of HyPer7, showing increased 405 nm excitation and decreased 488 nm excitation when oxidized (Fig. 1g). For comparison, we plotted the 405ex/488ex ratios. Interestingly, Pd-roGFP2 was insensitive to 1 or 2.5 mM H_2_O_2_ treatments (Fig. 1, h and i), which is markedly different from the saturated response of Pd-HyPer7. However, like Pd-HyPer7, Pd-roGFP2 was able to sense high levels of DTT and H_2_O_2_ (5 to 10 mM), albeit exhibiting only a maximum 0.3-fold change in ratio (Fig. 1, h and i), which corresponds to an 8-fold lower dynamic range revealed by Pd-HyPer7. Note that the full dynamic range of HyPer7 at plasmodesmata that we observed was from approximately 0.6 (in response to DTT) to 2.6 (in response to H_2_O_2_). Given the superior sensitivity and dynamic range of Pd-HyPer7, we selected it as an effective tool for subsequent studies.

### Plasmodesmata are highly sensitive to sub-millimolar H_2_O_2_ levels

**Figure 2:**
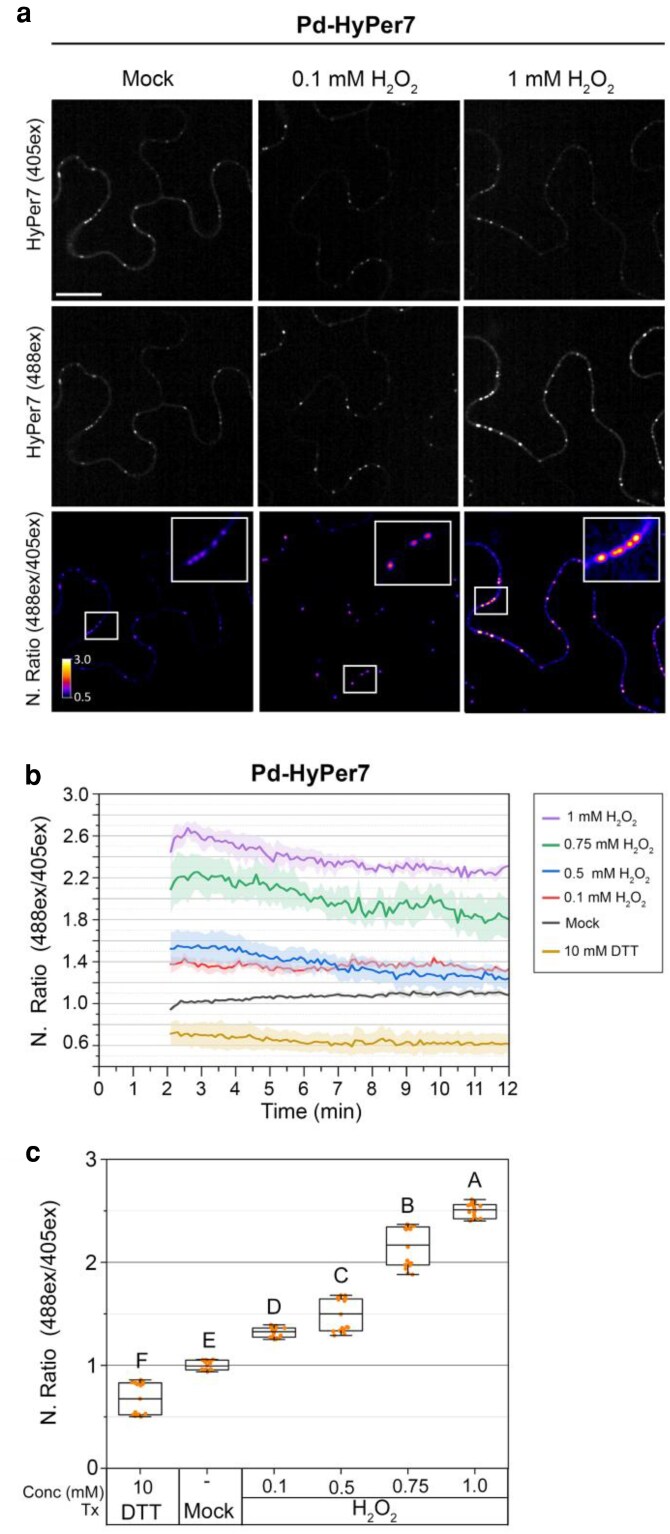
Pd-HyPer7 responses to sub-millimolar H_2_O_2_ levels. (a) Representative fluorescence images of Pd-HyPer7. Top panel, 405 nm excitation; middle panel, 488 nm excitation; and bottom panel, ratio image (488 nm ex./405 nm ex.). Images show responses to mock treatment (water), 0.1- and 1-mM H_2_O_2_ at 3-mpt. Scale bar, 20 µm. (b) Time-lapse ratio imaging over a 10-min period of Pd-HyPer7, following different treatments including varying concentrations of H_2_O_2_ at sub-millimolar levels. Shading represents SE. Data normalization and statistical analyses as in Fig. 1. (c) Box plot of Pd-HyPer7 ratios at 3-mpt across redox treatments. The graph represents data from a total of *n* = 6 images from at least four plants per treatment, combined from two replicate experiments.

As described earlier, the HyPer7 sensor at plasmodesmata became immediately saturated when plants were treated with H_2_O_2_ at millimolar levels (1 to 10 mM). This rapid saturation response raised the question whether plasmodesmata might be tuned to detect sub-millimolar, more physiological changes in H_2_O_2_ levels. To answer this question, we examined Pd-HyPer7's response to exogenously applied sub-millimolar H_2_O_2_ concentration (0.1 mM) to compare with its response to 1 mM treatment. Representative ratio images at 3-mpt showed an increased ratio after 0.1 mM H_2_O_2_ treatment compared to the mock, but this increase was much less pronounced than the 1 mM H_2_O_2_ treatment (Fig. 2a). This result clearly indicated that Pd-HyPer7 was sensitive enough to rapidly respond to exogenously applied H_2_O_2_ concentrations as low as 0.1 mM.

Based on this result, we then performed time-lapse measurements of Pd-HyPer7 after treating plants with a series of H_2_O_2_ concentrations ranging from 0.1 to 1 mM. The ratio image analysis of the time-lapse experiment revealed a distinctive pattern of a steep initial increase in ratio upon H_2_O_2_ treatments (Fig. 2b). For each sub-millimolar H_2_O_2_ concentration (0.1, 0.5, 0.75, and 1 mM), Pd-HyPer7 exhibited distinct oxidation states in a clear dose-dependent manner. Even 0.1 mM H_2_O_2_ treatment could induce an immediate 1.4-fold higher ratio than the mock at 3-mpt, which persisted throughout the time course (Fig. 2, b and c). These results demonstrate that plasmodesmata undergo rapid and dynamic redox changes in response to oxidative and reductive treatments.

### Subcellularly-targeted HyPer7 reporters show distinct responses to redox treatments

Plasmodesmata are continuous with both the cytosol and PM; however, it is unknown if they have similar or distinct redox characteristics. To compare redox dynamics of these compartments, we next generated two additional sensors, Cy (cytosol)- and PM-HyPer7. Cy-HyPer7 was similarly constructed to the original cytosolic HyPer7-NES (Pak et al. 2020; Ugalde et al. 2021b) by adding the same NES (nuclear-export sequence) to the C-terminal end of HyPer7, while PM-HyPer7 was produced by fusing HyPer7 to the C-terminal end of a PM-targeted PDLP5 mutant (Luna et al. 2023). Following confirmation of their correct localization (Figure S6a), we performed time-lapse experiments and quantitative analyses as described below.

**Figure 3:**
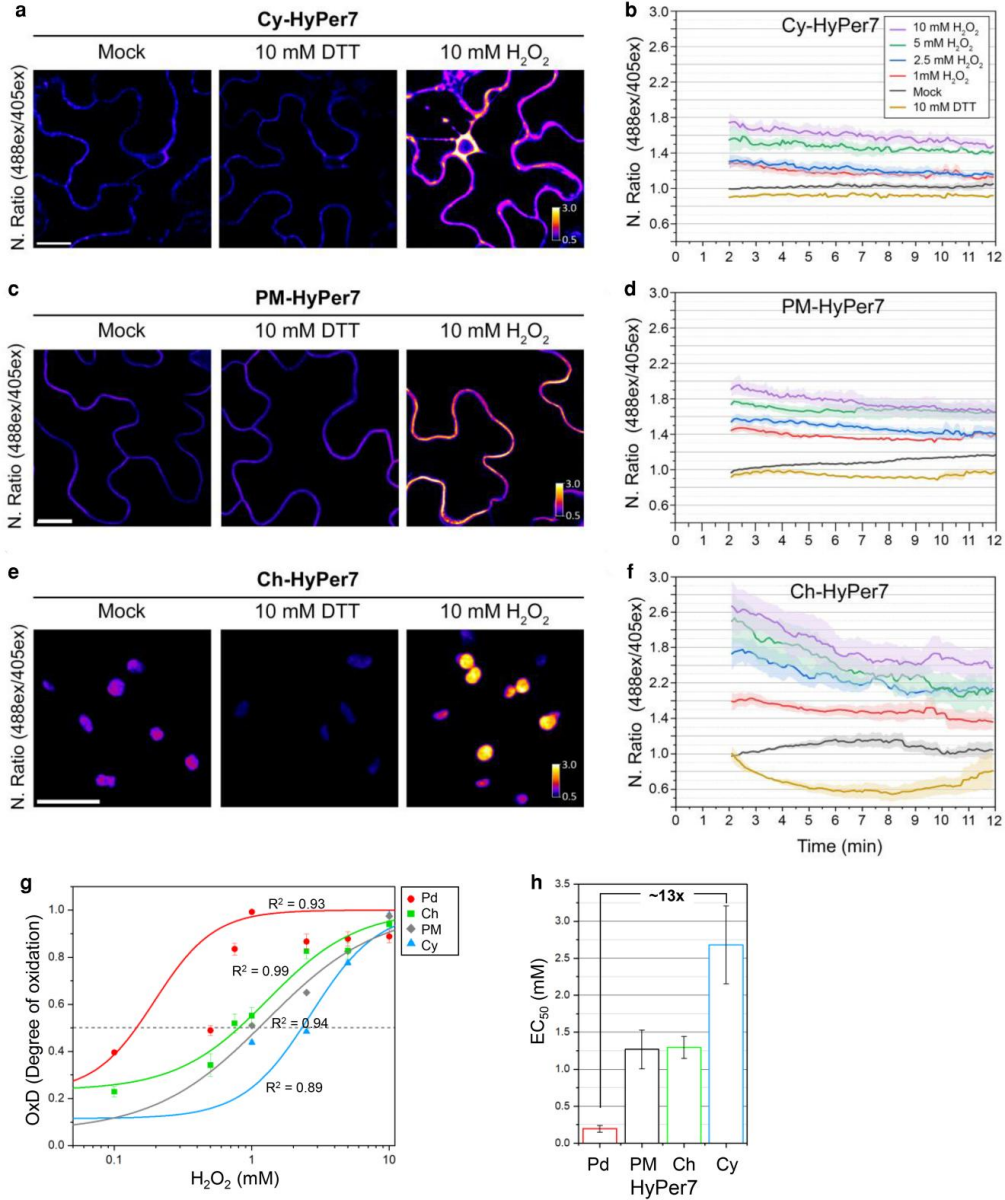
Pd-HyPer7 displays distinct response behaviors to exogenous redox treatments. (a), (c), and (e) Representative intensity ratio images of Cy-, PM-, and Ch-HyPer7 sensors in response to mock (water), 10 mM DTT, and 10 mM H_2_O_2_ treatments. Scale bars, 20 µm. (b), (d), and (f) Time-lapse changes of fluorescence intensity ratios over a 10-min period of subcellularly-localized HyPer7 sensors, each corresponding to panels A, C, or E, following different redox treatments including varying concentrations of H_2_O_2_. Each data point represents the mean ratio per image. Shading represents SE. Each time-lapse graph represents the ratio image data (*n* = 6 images collected using at least four plants per treatment) from two sets of independent replicate experiments. N. Ratio as in Fig. 1. (g) Dose-response curves showing degree of oxidation (OxD) as (*R*−*R*_min_)/(*R*_max_−R_min_), where *R*_min_ and *R*_max_ are the ratios at 10 mM DTT and 10 mM H_2_O_2_, respectively. Data are mean ± SEM (*n* = 18 to 42 images per concentration). Curves were fitted with the Hill equation. (h) EC_50_ values derived from Hill equation fits in (g). Error bars represent 95% confidence intervals of the fit. Ratio data at 3-mpt are from Figs 1e and 2b (Pd-HyPer7), 3B (Cy-HyPer7), and 3D (PM-HyPer7). Ratio data for Ch-HyPer7 were recollected under the same laser setting used for Cy-, PM-, or Pd-HyPer7 images at 3-mpt.

Representative ratio images showed that DTT (10 mM) treatment induced only a minor reduction of Cy-HyPer7 while exogenously applied H_2_O_2_ (10 mM) rapidly and substantially oxidized the sensor (Fig. 3a). Time-lapse analysis revealed that Cy-HyPer7 maintained a stable mean ratio over 10 min both in mock and DTT treated leaves with the latter showing approximately 10% reduction in mean ratio relative to the mock (Fig. 3b). In contrast, H_2_O_2_ treatments induced rapid rises in mean ratios of Cy-HyPer7 in a dose-dependent manner, exhibiting approximately 30% (1 and 2.5 mM H_2_O_2_), 50% (5 mM), and 70% (10 mM) increases in initial mean ratios relative to mock, and 10% to 20% overall decline over time. Notably, high millimolar concentrations of H_2_O_2_ did not saturate Cy-HyPer7, consistent with strong cytosolic redox buffering capacity and/or limited accumulation of applied H_2_O_2_ in the cytosolic sensor environment. The PM-HyPer7 responses were overall comparable to those of the Cy-HyPer7 (Fig. 3, c and d), as expected given the close proximity of the PM reporter to the cytosol-facing cellular environment.

To extend the comparison to another key redox-active compartment and a major cellular source of H_2_O_2_ (Asada 2006; Mignolet-Spruyt et al. 2016; Smirnoff and Arnaud 2019), we also investigated the chloroplast. To this end, we produced chloroplast-targeted HyPer7 (Ch-HyPer7) by fusing a transit peptide at the N-terminus of HyPer7 and confirmed its correct targeting (Figure S6a). To minimize chloroplasts’ high susceptibility to light-induced ROS production (Exposito-Rodriguez et al. 2017; Dopp et al. 2023) (Figure S7), we performed time-lapse experiments under a reduced laser power (Table S1) (Fig. 3, e and f). Ch-HyPer7 showed rapid, dose-dependent oxidation to H_2_O_2_ with subsequent partial recovery over time (Fig. 3f). H_2_O_2_ treatments (1 to 10 mM) rapidly produced a 1.4- to 2.6-fold increase in Ch-HyPer7 ratio compared to mock-treated samples, followed by partial recovery over time (Fig. 3f). DTT (10 mM) treatment, on the other hand, produced a slower but substantial reduction, reaching a 40% reduction by 6-mpt followed by a gradual recovery phase, eventually returning toward the initial 3-mpt value. These results highlight chloroplasts’ dynamic redox regulation, consistent with chloroplasts’ robust redox homeostasis (Exposito-Rodriguez et al. 2017; Dopp et al. 2023).

To compare the operational responsiveness of the four targeted HyPer7 reporters to exogenous H_2_O_2_ treatments, ratio values were then converted to degree of oxidation (OxD), adapted from the roGFP2 calibration framework (Schwarzlander et al. 2008), which was recently applied to HyPer and HyPer7 (Booth et al. 2021; Young et al. 2024; Huang et al. 2025). Accordingly, OxD values were calculated as (*R*−*R*_min_)/(*R*_max_−*R*_min_), where *R*_min_ and *R*_max_ are set to the ratios at 10 mM DTT and 10 mM H_2_O_2_, respectively, and the resulting OxD dose-response data were fitted with the Hill equation (Fig. 3g). These treatments are used here as operational calibration extremes for normalization. For Ch-HyPer7, we acquired its ratio images at 3-mpt in leaves treated with stepwise H_2_O_2_ (0.1 to 10 mM) and DTT (10 mM) under identical laser power settings used for other compartments (Table S1). We treat the OxD values and apparent half-maximal oxidation (EC_50_) as operational measures of sensor response under exogenous treatment conditions, not absolute intracellular H_2_O_2_ concentrations. These parameters are therefore used to compare relative reporter responsiveness rather than to infer absolute in vivo H_2_O_2_ concentrations among compartments. Before quantitative comparison across targeted reporters, we examined whether basal HyPer7 ratios were dependent on local reporter concentrations, given that in vitro assays have shown concentration-dependent HyPer7 behavior (Pak et al. 2020). We used total fluorescence intensity within each measured ROI as an approximation for the reporter concentration and plotted this value against the corresponding 488e/405e ratio collected under mock conditions (Figure S6b). Each data point represents an individual plasmodesmal spot or similarly sized ROI measured from chloroplast or cytosol reporter images. Linear regression analysis of the resulting plots did not reveal a systematic relationship between ROI fluorescence intensity and ratio across the measured intensity range. This result indicates that local HyPer7 intensity does not produce a simple directional bias in the mean ratio, consistent with previous reports (Dopp et al. 2023). However, we acknowledge that we cannot exclude concentration-dependent effects of HyPer7 and therefore interpret intercompartmental comparisons cautiously as operational comparisons of HyPer7 responses under defined experimental conditions.

The OxD analysis showed that Pd-HyPer7 under our experimental conditions had the lowest apparent EC_50_ of approximately 0.2 mM, followed by Ch- and PM-HyPer7 with 1.1 and 1.3 mM, respectively. In contrast, Cy-HyPer7 required a substantially higher concentration (∼2.7 mM) to achieve a comparable oxidation, corresponding to approximately 13-fold higher apparent EC_50_ than Pd-HyPer7 (Fig. 3h). Notably, while both Pd- and Ch-HyPer7 responded to sub-millimolar H_2_O_2_, Pd-HyPer7 exhibited a steep, switch-like response, reaching near-saturation by 1 mM, whereas Ch-HyPer7 showed a more gradual rise across a broader concentration range (Fig. 3g). Together these data show that Pd-HyPer7 has a high apparent responsiveness to exogenous H_2_O_2_ treatments and a response profile distinguishable from the other targeted HyPer7 reporters under the conditions tested.

### Subcellularly-targeted APX effectively reduces plasmodesmata

Having found that plasmodesmata display distinct redox response characteristics, we next asked whether targeted H_2_O_2_ scavenging could modulate the Pd-HyPer7 readout. In order to achieve targeted redox modulation beyond relying on nonspecific chemical reductants like DTT, we expressed *Arabidopsis* ascorbate peroxidase (APX) 1, known to scavenge H_2_O_2_ in the cytosol (Nedo et al. 2024), in chloroplasts, cytosol, or plasmodesmata. (Constructs included mKate2 for visualization (Figure S8)). Because redox production/buffering and biosensor behavior differ by compartment, we interpret APX effects within each compartment rather than by direct cross-compartment comparison.

**Figure 4:**
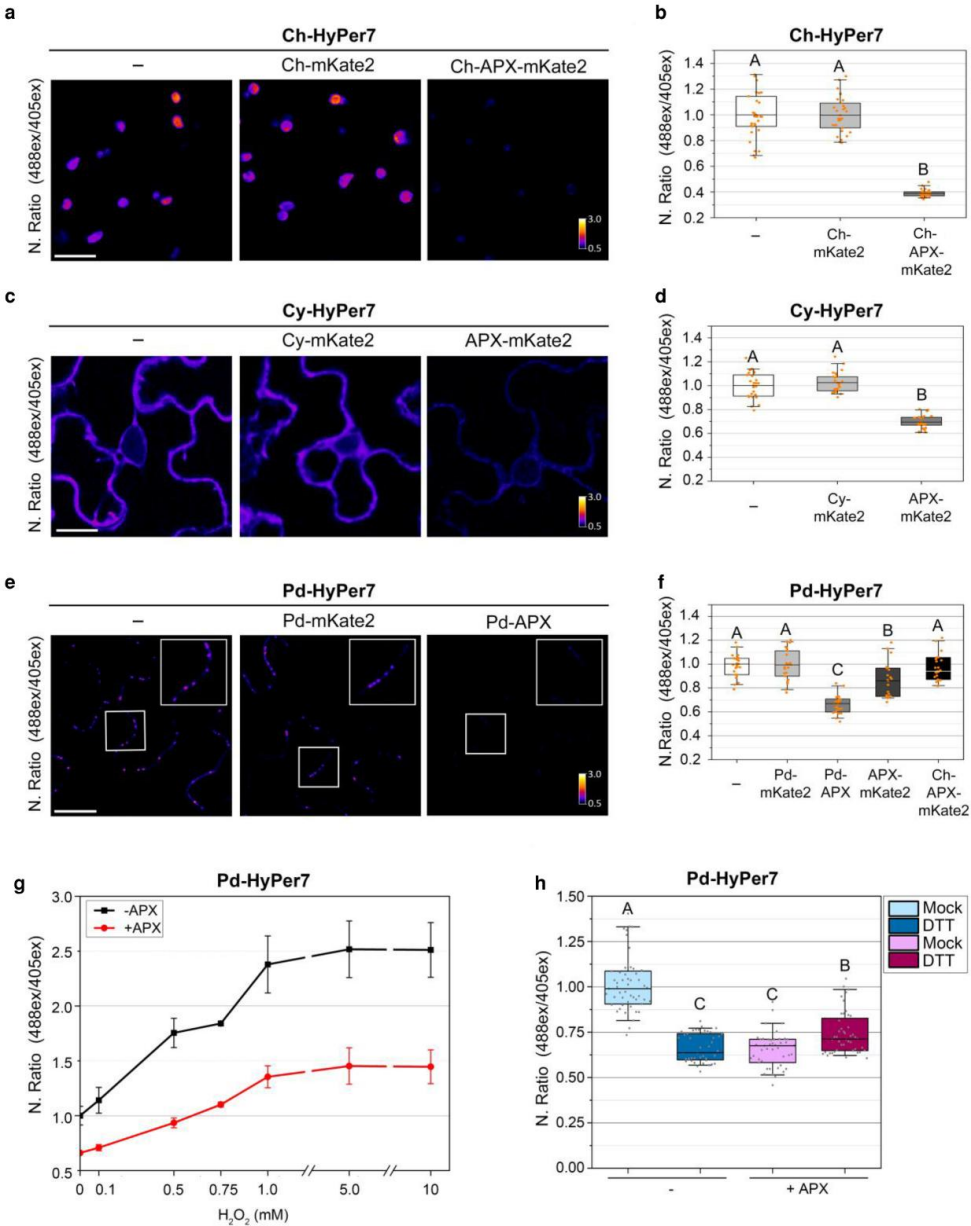
Plasmodesmata can be effectively reduced by pd-APX. (a), (c), and (e) Representative fluorescence ratio (488 nm ex./405 nm ex.) images of Ch-, Cy-, and Pd-HyPer7 sensors in the absence (−) or presence of mock (mKate2) or APX co-expression, each targeted to the same compartment as the sensor (for the cytosolic APX, APX-mKate2 localized primarily to the cytosol with weak nuclear signal). (b) and (d) Box plots of HyPer7 ratios corresponding to panels, A and C, respectively. (f) Box plot comparing Pd-HyPer7 ratios in the absence (−) of ectopically expressed APX or the presence of APX targeted to plasmodesmata, cytosol, or chloroplasts. (g) Line graph showing Pd-HyPer7 response to H_2_O_2_ attenuated by Pd-targeted APX. Each data set was collected from a total of *n* = 35 to 50 images using at least eight plants per treatment from two independent experiments. (h) Box plot comparing DTT effects on Pd-HyPer7 ratios with and without Pd-targeted APX. Data were collected for each treatment from a total of *n* > 20 images (at 3-mpt) using at least four plants from at least two independent experiments. Data are normalized to the mean ratio of mock-treated samples. Statistical analyses for panels B, D, and F were as in Fig. 1. Statistical analysis for panel H was performed using two-way ANOVA followed by Tukey's post-hoc test (*α* = 0.05) with different letters indicating significant differences. The interaction between APX and DTT treatment was significant (*P* < 0.0001).

Representative ratio images showed that while expression of Ch-mKate2 control had no effect on chloroplast redox state, Ch-APX-mKate2 substantially reduced the ratio values as indicated by the clear shift from magenta to blue color (Fig. 4a). Quantitative analysis (Fig. 4b) revealed that Ch-APX achieved a markedly stronger reduction of chloroplasts than previously observed with DTT treatment (see Fig. 3f), demonstrating the high effectiveness of this compartment-specific approach. Similarly, the presence of APX in the cytosol could also reduce the cytosol more effectively (Fig. 4, c and d) than DTT. Consistent with these findings, Pd-targeted APX also reduced plasmodesmata relative to control treatments (Fig. 4, e and f). Note that untagged Pd-APX showed slightly higher efficacy than the mKate2-tagged version (Figure S9), likely due to its smaller size. Interestingly, the co-expression of cytosolic APX exhibited a slight reductive effect on Pd-HyPer7 while chloroplastic APX had no effect (Fig. 4f). Furthermore, Pd-APX proved highly effective at suppressing H_2_O_2_-dependent oxidation of plasmodesmata across the whole range of concentrations used in our experiments. For example, when challenged with exogenous H_2_O_2_, ranging from sub-millimolar to high millimolar concentrations, control plants showed pronounced oxidation with the sensor ratio reaching saturation around a 2.5 ratio at 3-mpt, while Pd-APX-expressing plants maintained significantly lower ratios, remaining below 1.5 even at the highest H_2_O_2_ concentration (Fig. 4g). These results demonstrate Pd-targeted APX as an effective tool for modulating plasmodesmal redox state.

Notably, Pd-APX achieved reduction levels comparable to those induced by DTT treatment in mock-treated plants (Fig. 4h), prompting us to ask whether combining these two approaches might further reduce plasmodesmata. To better understand the limits of plasmodesmal reduction, we, therefore, examined how plants expressing Pd-APX would respond to additional reductive stress from DTT treatment. Intriguingly, while DTT reduced the Pd-HyPer7 ratio in control plants, this treatment significantly increased the ratio in plants expressing Pd-APX (Fig. 4h). This counterintuitive result suggests that Pd-APX achieves maximal physiological reduction of plasmodesmata, beyond which additional reductants paradoxically induce oxidative stress.

### An SA-deficient state alters compartment-specific redox responses

**Figure 5:**
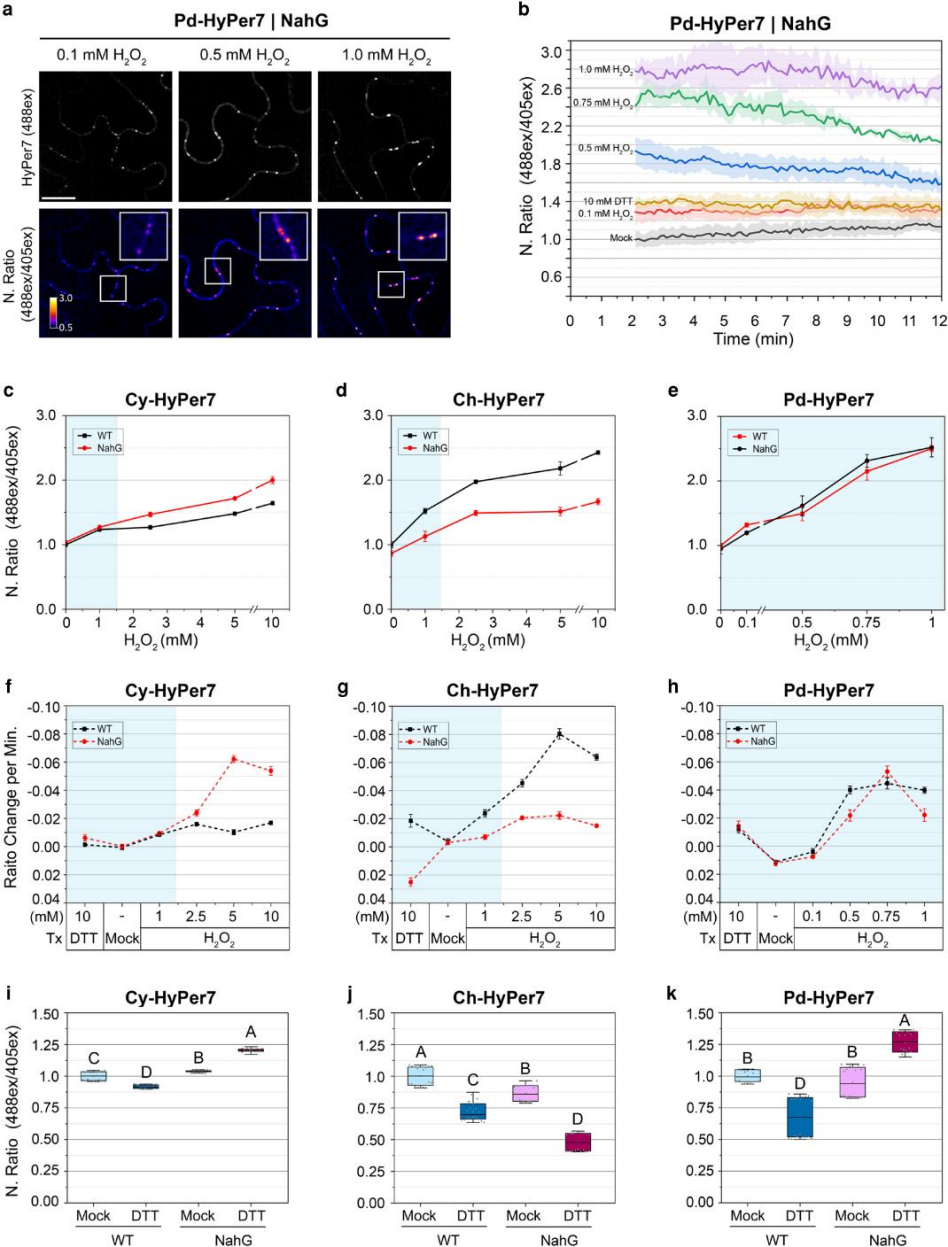
Redox responses and dynamics are altered in *NahG.* (a) Representative ratio images of Pd-HyPer7 in *NahG*. Top panel, 488 nm excitation and bottom panel, ratio image (488 nm ex./405 nm ex.). Images show responses to H_2_O_2_ (0.1, 0.5, and 1-mM) at 3-mpt (mock is shown in the time-course in panel B). Ratio values are represented by pseudocolor Fire LUT scaled from 0.5 to 3.0. (b) Time-lapse of Pd-HyPer7 in *NahG* under different redox conditions. Shading represents SE. Data were collected for each treatment from a total of *n* = 6 images using at least four plants from two independent experiments and normalized to the mean value of mock-treated samples from the first 1-min imaging interval (corresponding to 3-mpt). (c) to (e) Line graphs comparing *NahG* to WT responses to H_2_O_2_, which show HyPer7 oxidation enhanced in the cytosol (c), dampened in chloroplasts (d), and normal at plasmodesmata (e). (f) to (h) Line graphs comparing temporal redox dynamics (measured as ratio changes between early [3 min] and late [10 min] time points) in *NahG* to WT across treatments (DTT, mock, and varying concentrations of H_2_O_2_). Negative *Y*-axis values indicate decreasing ratio (recovery from oxidation) and positive values an increasing ratio (progressive oxidation). For ease of visualizing recovery, the *y*-axis is plotted with negative values upward (faster recovery from oxidation) and positive values downward (progressive oxidation). (i) to (k) Box plots comparing HyPer7 ratios in *NahG* to WT with and without DTT treatment. The cytosol (i) and plasmodesmata (k) show unexpected oxidative response to DTT in *NahG.* Data extracted from a series of time-lapse images were normalized to the mean value of mock-treated *NahG* or WT samples at 3-mpt (the full time-lapse data for the cytosol and chloroplasts are shown in Figures S13 and S15). All data were collected from a total of *n* > 20 images at 3-mpt using at least six plants per treatment from two independent experiments. Error bars represent SE. Statistical analyses were performed using two-way ANOVA followed by Tukey's post-hoc test (*α* = 0.05). The interaction between genotype and DTT treatment was significant (*P* < 0.0001). Different letters indicate significant differences between groups.

To gain further insight into the compartment-specific redox dynamics, particularly at plasmodesmata, we employed our suite of subcellularly targeted HyPer7 sensors for comparative analyses between WT and *NahG N. benthamiana* plants. NahG is a bacterial enzyme that breaks down SA to catechol, and hence, transgenic plants expressing *NahG* are depleted in SA (Friedrich et al. 1995). Previously, we discovered that transgenic *NahG Arabidopsis* plants, as well as SA biosynthetic mutants, had enhanced plasmodesmal permeability and that SA and ROS independently modulate plasmodesmal permeability through distinct signaling pathways (Wang et al. 2013; Cui and Lee 2016). To use HyPer7 to further investigate redox dynamics at plasmodesmata in *NahG* plants, we first verified that *NahG* increases plasmodesmal permeability in *N. benthamiana* using GFP movement assays. Consistent with our previous finding in *Arabidopsis NahG* plants (Wang et al. 2013), the result showed that GFP movement in *NahG* was more extensive compared to WT plants (Statistical analysis showed that *NahG* plants exhibit 2.6-fold higher odds of GFP spreading to more cells compared to WT [ordinal logistic regression, *P* < 0.0001]) (Figure S10). Then, we expressed Pd-HyPer7 in *NahG* plants and examined its response to H_2_O_2_. When treated with increasing concentrations of H_2_O_2_ (0.1 to 1.0 mM), punctate structures in Pd-HyPer7 ratio images showed progressive increases, reflecting local oxidation states of plasmodesmata (Fig. 5a, ratiometric images in lower panels).

Time-course ratio analyses revealed H_2_O_2_ treatments induced sustained oxidation with gradual adaptation over time in *NahG* plasmodesmata (Fig. 5b and Figure S11), which was a similar response observed in WT plants (Fig. 2b). The apparently normal H_2_O_2_ response at *NahG* plasmodesmata, despite their enhanced plasmodesmal permeability, corroborates our previous finding that SA regulates plasmodesmal permeability independently of redox signaling (Cui and Lee 2016). Indeed, when we treated WT plants with SA or its analog benzothiadiazole for 30, 60, or 120 min, these treatments did not alter the redox state at plasmodesmata, demonstrating that SA does not alter plasmodesmal redox states under normal conditions (Figure S12).

Next, we investigated if the lack of SA in *NahG* impacts redox responses in other subcellular compartments, including the cytosol, PM, and chloroplasts, using HyPer7 targeted to these compartments and performed comparative H_2_O_2_ dose-response measurements in time-course ratio imaging. The HyPer7 responses to treatments in the cytosol (Figure S13) were indistinguishable from the PM (Figure S14), and thus, we chose to focus on the cytosolic data for further analyses. Compared to WT, the *NahG* H_2_O_2_ dose-response curves showed enhanced cytosolic response to higher concentrations of H_2_O_2_ (2.5 to 10 mM; Fig. 5c) and a greatly dampened chloroplastic response to all concentrations of H_2_O_2_ (Fig. 5d and Figure S15). In contrast, the plasmodesmal responses remained similar between WT and *NahG* at low concentrations of H_2_O_2_ (0.1 to 1 mM; Fig. 5e). Given that these data revealed distinct patterns across compartments, we decided to further investigate these differences by analyzing the rate of ratio change between the early (3-mpt) and late (10-mpt) time points using the data extracted from the time-course ratio imaging. In *NahG* plants, the cytosolic HyPer7 ratio decreased more rapidly (more negative rate values) in plants treated with higher H_2_O_2_ concentrations (5 to 10 mM; Fig. 5f), indicating faster recovery from initial oxidation. In contrast, *NahG* chloroplasts showed slower ratio decline (less negative rate values) across all H_2_O_2_ concentrations (Fig. 5g), suggesting dampened recovery dynamics. The response kinetics of plasmodesmata in *NahG* treated with sub-millimolar H_2_O_2_ were highly dynamic and almost identical to WT (Fig. 5h).

To further investigate compartment-specific redox responses, we analyzed the statistical differences in HyPer7 ratios between WT and *NahG* plants following mock or DTT treatment using two-way ANOVA with Tukey's post-hoc test (Fig. 5, i to k). Under mock conditions, the *NahG* cytosol showed a statistically significant but modest elevation (∼4%; Fig. 5i), whereas the *NahG* chloroplast ratio was substantially lower (Fig. 5j). In contrast, plasmodesmata did not differ from WT under mock conditions (Fig. 5k). However, the most striking finding was that WT and *NahG* responded to DTT in opposite directions. While all three WT compartments showed the expected reduction in response to DTT, both the *NahG* cytosol and plasmodesmata exhibited a rapid paradoxical oxidative response. Notably, plasmodesmata showed the most dramatic response reversal despite having no difference under mock conditions between genotypes. At plasmodesmata, this result recapitulated our earlier observation with Pd-targeted APX (Fig. 4h), and the oxidative spike was comparable in magnitude to the *NahG* plasmodesmal oxidation induced by 0.1 mM H_2_O_2_ (Fig. 5b). These findings indicate an enhanced sensitivity to reductive stress in a compartment-specific manner, suggesting that *NahG* plasmodesmata operate under altered redox regulatory mechanisms that render them particularly susceptible to reductive perturbation.

### Both cold and mechanical wounding induce rapid oxidation at plasmodesmata

**Figure 6:**
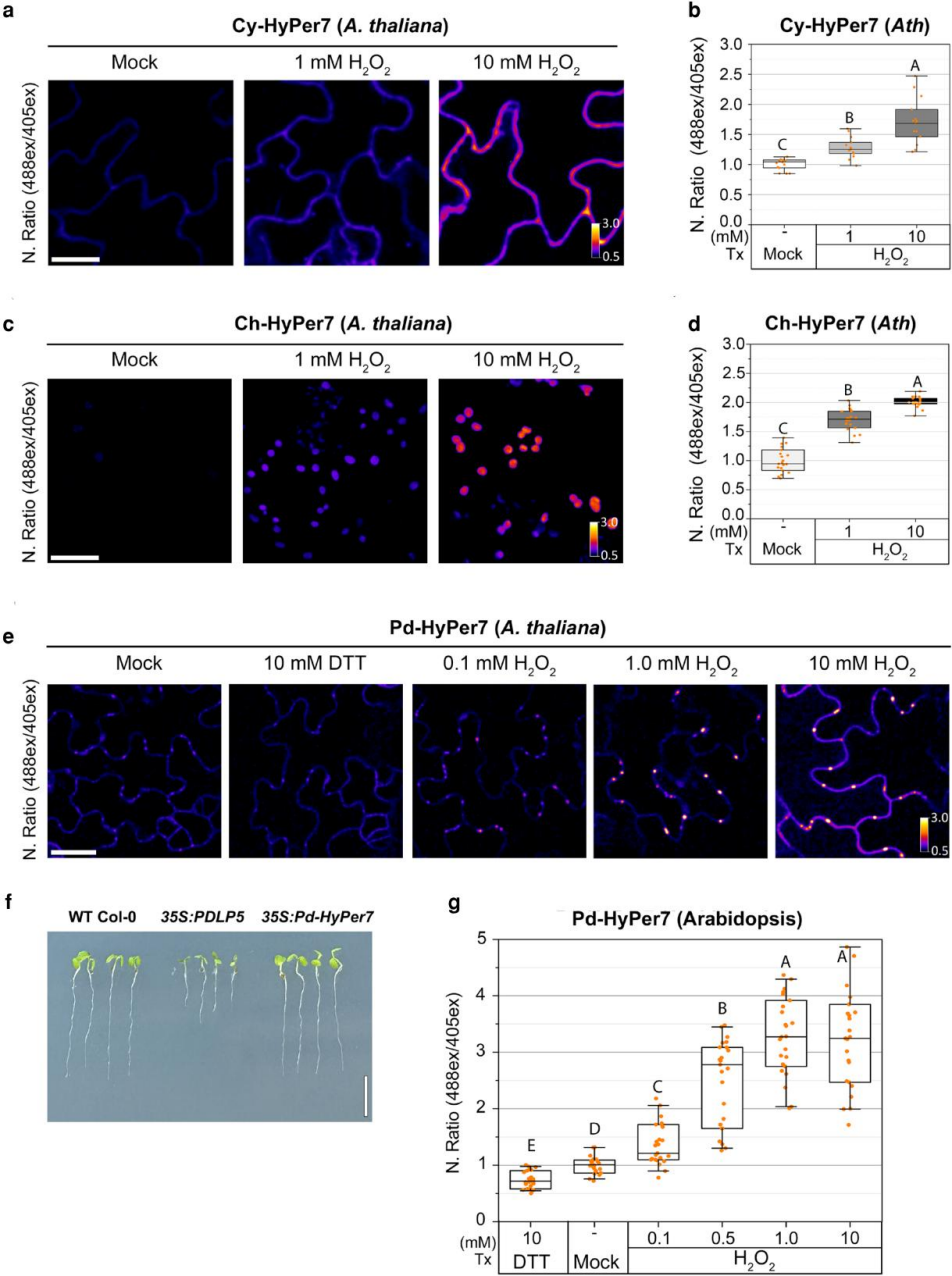
HyPer7 reporters show distinct sensitivities to redox treatments in intact *Arabidopsis* seedlings. (a) and (b) Representative ratio images and corresponding box plot of Cy-HyPer7 in cotyledon epidermal cells of 7-day-old seedlings treated with mock, 1, and 10 mM H_2_O_2_. Ratio values (488 nm ex./405 nm ex.) are represented by pseudocolor Fire LUT scaled from 0.5 to 3.0. (c) and (d) Representative ratio images and corresponding box plot of Ch-HyPer7. (e) Representative fluorescence ratio images of Pd-HyPer7 showing responses to mock, 10 mM DTT, 0.1, 1, and 10 mM H_2_O_2_ treatments. (f) Image of WT Col-0, *35S::PDLP5,* and *35S::Pd-HyPer7* (homozygous line #T3 to 27) seedlings grown vertically on ½ MS agar plates. The picture was taken at 7 d post-imbibition. The *35S::PDLP5* line is originally reported by Lee et al. (2011). (g) Box plot of Pd-HyPer7 ratios across treatments. Scale bars—20 μm (a), (c), and (e) and 0.5 mm (f). Data were collected from *n* = 20 images using at least 10 seedlings per treatment from two independent experiments. Data are normalized to the mean ratio of mock-treated seedlings.

To enable monitoring ROS dynamics in intact seedlings, we generated stable transgenic *Arabidopsis* lines focusing on Ch-, Cy, and Pd-HyPer7 reporters. Treatments were performed using 7-day-old seedlings by gentle syringe infiltration of a whole seedling with water mock control or solutions containing H_2_O_2_ or DTT, immediately followed by ratio imaging of cotyledon epidermal cells. Correct localization, functionality, and responsiveness of the three HyPer7 reporters in *Arabidopsis* seedlings (Fig. 6 and Figures S16 to S18) were comparable to that of *N. benthamiana* leaves. In our previous study, we reported that expressing *PDLP5* at high levels causes stunted growth (Lee et al. 2011). Considering that this might compromise the utility of the Pd-HyPer7 reporter lines, we isolated those lines that express the sensor just high enough for microscopic detection without causing visible growth phenotypes. Thus identified T2 or T3 seedlings of one of the lines, *35S:Pd-HyPer7* (line#27) (Fig. 6f and Figure S18), were used for further studies. Notably, this reporter line exhibited similar gradual responsiveness to sub-millimolar H_2_O_2_ treatments with slightly higher dynamic ranges, reaching over 3-fold increase in ratio upon 1 or 10 mM H_2_O_2_ treatment (Fig. 6g). We cannot fully exclude PDLP5-dependent effects on plasmodesmal physiology; however, the transient mTM control and lack of growth defects support that the observed HyPer7 dynamics primarily reflect compartmental redox responsiveness.

**Figure 7:**
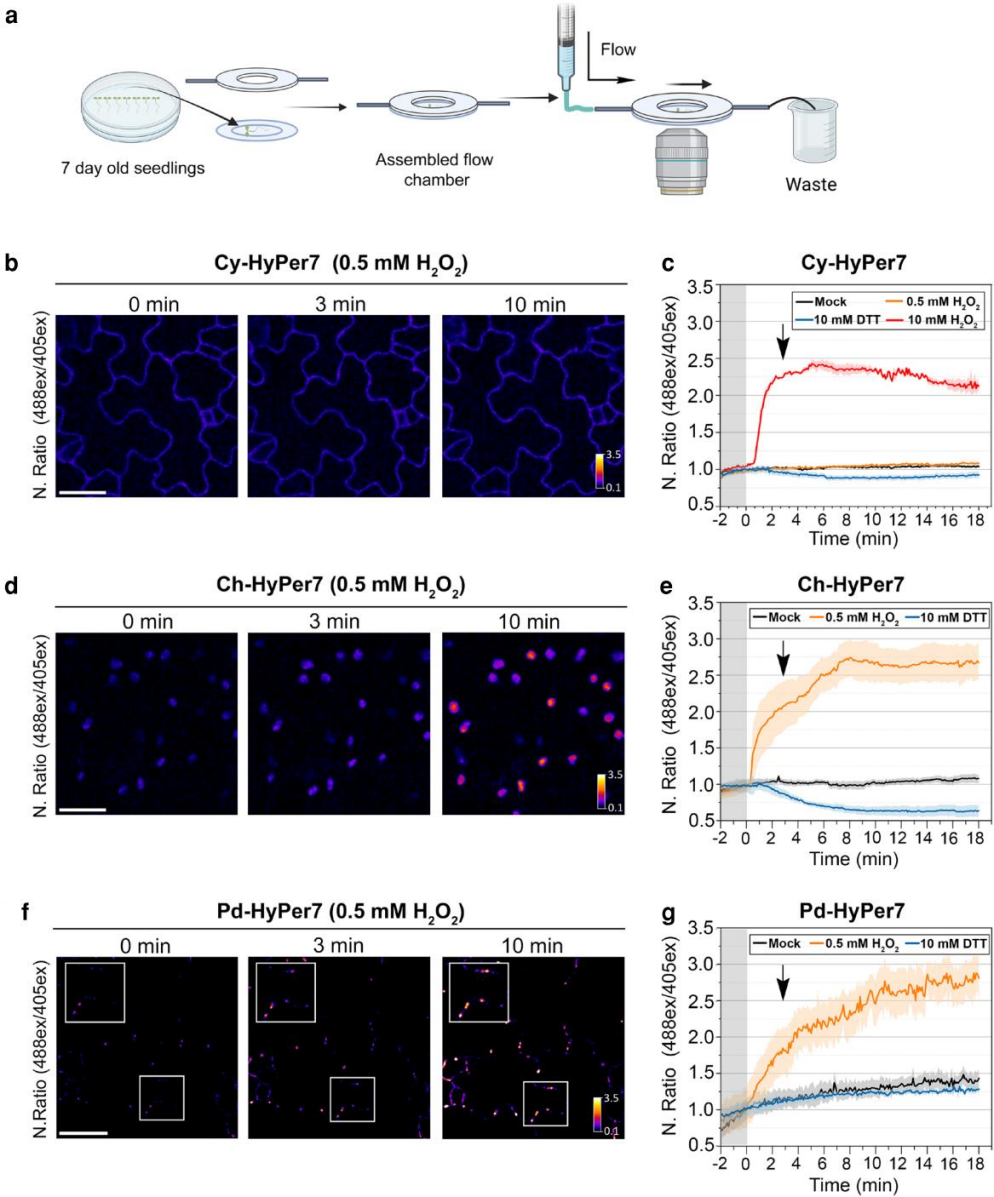
Real-time monitoring of redox dynamics using a flow chamber system. (a) Schematic diagram of the flow chamber setup showing 7-day-old seedlings mounted in an assembled flow chamber connected to a flow delivery system with waste collection. Created in BioRender. (b), (d), and (f) Representative ratio images of Cy-, Ch-, and Pd-HyPer7 during 0.5 mM H_2_O_2_ flow treatment at 0, 3, and 10 min. Insets are enlarged areas (panel f). Scale bars—20 μm. (c), (e), and (g) Time-lapse analysis of Cy-HyPer7 (c), Ch-HyPer7 (e), and Pd-HyPer7 (g) responses to mock, 0.5 mM H_2_O_2_ (and 10 mM H_2_O_2_ for Cy-HyPer7), and 10 mM DTT treatments. Shaded areas, baseline recording period; arrows, 3-mpt. Shading represents SE. Each time-course analysis represents data from *n* = 3 to 5 seedlings per treatment. Ratio values are normalized to each sensor's mock at the 0 time point collected in the same experimental session.

Next, we examined real-time redox dynamics in *Arabidopsis* seedlings using a flow chamber system (Fig. 7a and Figure S19). Intact seedlings were mounted in a chamber constructed from adhesive spacer sheets, allowing continuous flow of treatment solutions over the seedling while enabling real-time imaging of cotyledon cells. Solutions were delivered through tubing connected to a gravity flow syringe system (6.8 ml/min), providing consistent, reproducible flow between different treatments and replicates during time-course experiments. As expected from its high buffering capacity, the cytosol showed no detectable changes in Cy-HyPer7 ratio to 0.5 mM H_2_O_2_ treatment, with the ratio remaining stable throughout the time course (Fig. 7, b and c). In contrast, 10 mM H_2_O_2_ treatment triggered an immediate and dramatic increase in ratio, reaching a plateau of approximately 2.5-fold within 2 to 3 min and maintaining this elevated level throughout the treatment period (Fig. 7c and Video S1). Additionally, 10 mM DTT treatment produced a slight reduction. In contrast, both Ch- and Pd-HyPer7 showed rapid oxidation at 0.5 mM H_2_O_2_ (Fig. 7, d to g and Videos S2 and S3). Interestingly, while both Cy- and Ch-HyPer7 remained stable under mock (water) flow treatment for over 20 min, Pd-HyPer7 showed a slight but steady increase in ratio with mock treatment, indicating an oxidation response under our flow conditions. Also, these sensors responded to 10 mM DTT with Ch-HyPer7 showing a more pronounced response. These results confirmed that our flow system is functional and enables real-time monitoring of treatment responses in a continuous sequence.

**Figure 8:**
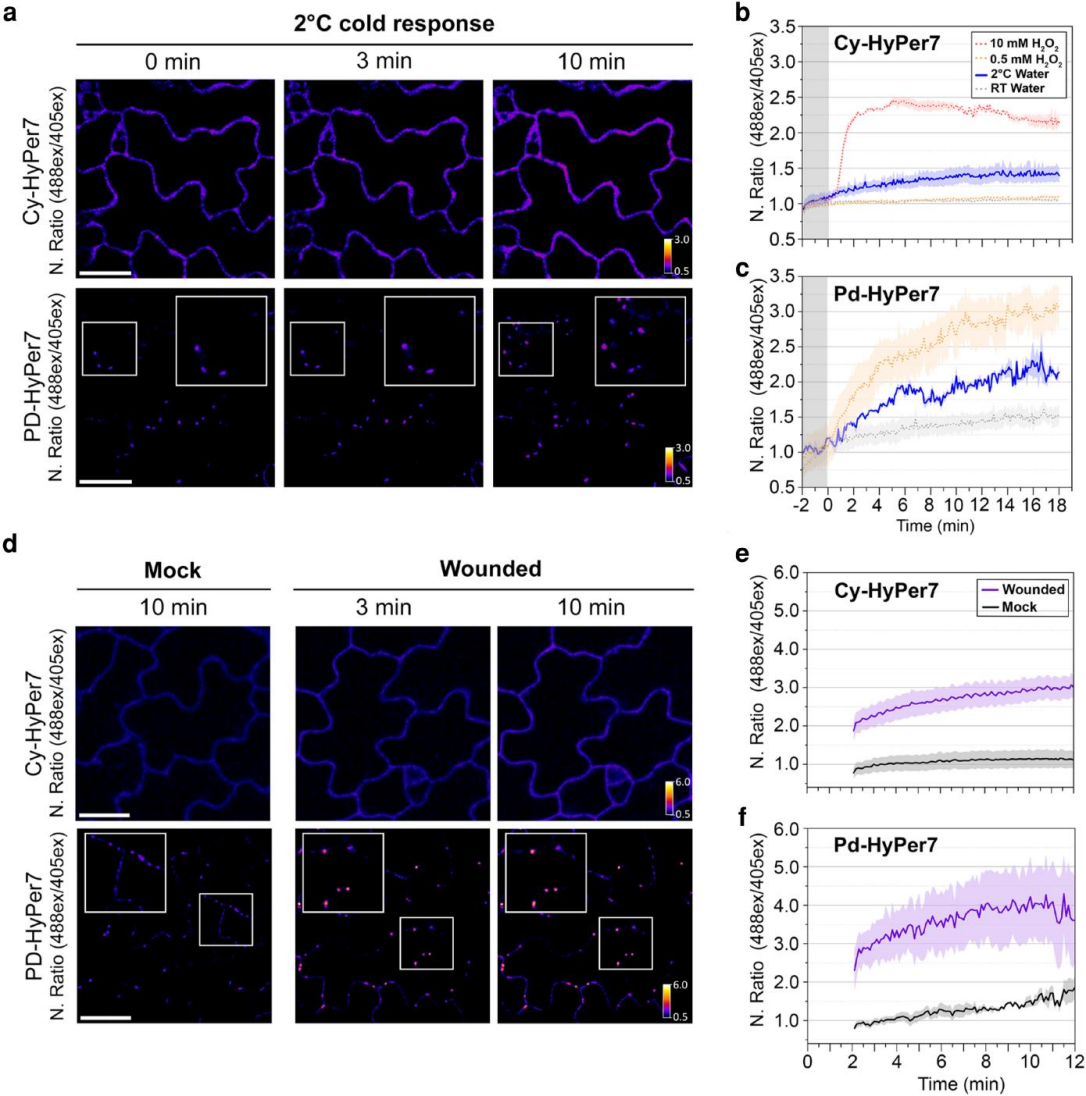
Cold stress and mechanical wounding induce rapid oxidation responses at plasmodesmata. (a) Representative fluorescence ratio images of Cy-HyPer7 (top panels) and Pd-HyPer7 (bottom panels) during 2 °C cold water flow treatment at 0, 3, and 10 min. Insets are enlarged areas. Scale bars—20 μm. (b) and (c) Time-course analyses of Cy-HyPer7 (b) and Pd-HyPer7 (c) responses to cold treatment. Shaded area indicates baseline recording period before treatment. Treatment starts at time 0. Mock and H_2_O_2_ response data from Fig. 7 are overlaid for comparison. Data were normalized to the mean value of 23 °C water-treated samples from the 1-minute baseline period preceding treatment (single z-stack images, captured at 6-second intervals). (d) Representative fluorescence ratio images of Cy- and Pd-HyPer7 following mechanical wounding at 3- and 10-mpw (minutes post-wounding). Unwounded mock sample imaged at 10-mpw is shown for comparison. Insets are enlarged areas. Scale bars—20 μm. (e) and (f) Time-lapse analyses of Cy-HyPer7 (e) and Pd-HyPer7 (f) responses to mechanical wounding. Images acquired as 10 z-stack images, using a 2 μm interval, captured at 6-second intervals under identical laser settings for the cytosol and plasmodesmata. Data are normalized to the mean value of mock-treated samples at 3-mpw. Data represent *n* = 6 independent seedlings per treatment. Shading represents SE.

To further demonstrate real-time plasmodesmal redox responses under biological stress conditions, we treated Arabidopsis seedlings with cold stress. Cold stress is known to alter plasmodesmal permeability (Bilska and Sowinski 2010; Murata et al. 2025), but whether this response is rapid and involves changes in redox states remained unknown. We examined the cytosolic response to cold stress by switching from room temperature (RT, 23 °C) water to ice-cold water (2 °C) flow. Surprisingly, cold treatment induced a steady increase in Cy-HyPer7 ratio, reaching a maximum 0.5-fold increase within 10 to 12 min (Fig. 8, a and b and Video S4). This result indicates that H_2_O_2_ levels rise gradually in the cytosol under cold temperature stress. Pd-HyPer7 also exhibited a clear oxidation response over the 10 to 12 min cold exposure window (Fig. 8, a and c and Video S3). This cold-induced plasmodesmal response was intermediate between the RT water response and the 0.5 mM H_2_O_2_ treatment, demonstrating that cold stress generates a moderate, but physiologically relevant, level of oxidative stress at plasmodesmata.

Next, we examined the real-time responses to mechanical wounding in both the cytosol and plasmodesmata. We have previously shown that plasmodesmata close upon mechanical wounding and that this response is mediated by H_2_O_2_ (Cui and Lee 2016). However, the ROS states and dynamics at plasmodesmata during plant wounding responses remained unknown, since plasmodesmal-localized H_2_O_2_ measurements were not previously feasible even under normal conditions. To visualize changes in H_2_O_2_ levels upon wounding, one cotyledon of an intact, mounted 7-day-old transgenic seedling was mechanically wounded with a sharp scalpel and immediately imaged. Wounding rapidly increased HyPer7 ratios both in the cytosol (Fig. 8, d and e and Video S5) and plasmodesmata (Fig. 8, d and f and Video S6). The magnitude of the Pd-HyPer7 oxidation approaches the upper range of Pd-HyPer7 oxidation observed under high-dose exogenous H_2_O_2_ acquired using the same imaging settings (see Fig. 6, e and g). Importantly, this comparison is qualitative and does not imply an equivalent intracellular H_2_O_2_ concentration, because wounding involves multiple signals and the effective oxidant exposure at plasmodesmata is not directly measurable from exogenous dose alone.

### Mechanical wounding reveals distinct redox dynamics between the cytosol and plasmodesmata

**Figure 9:**
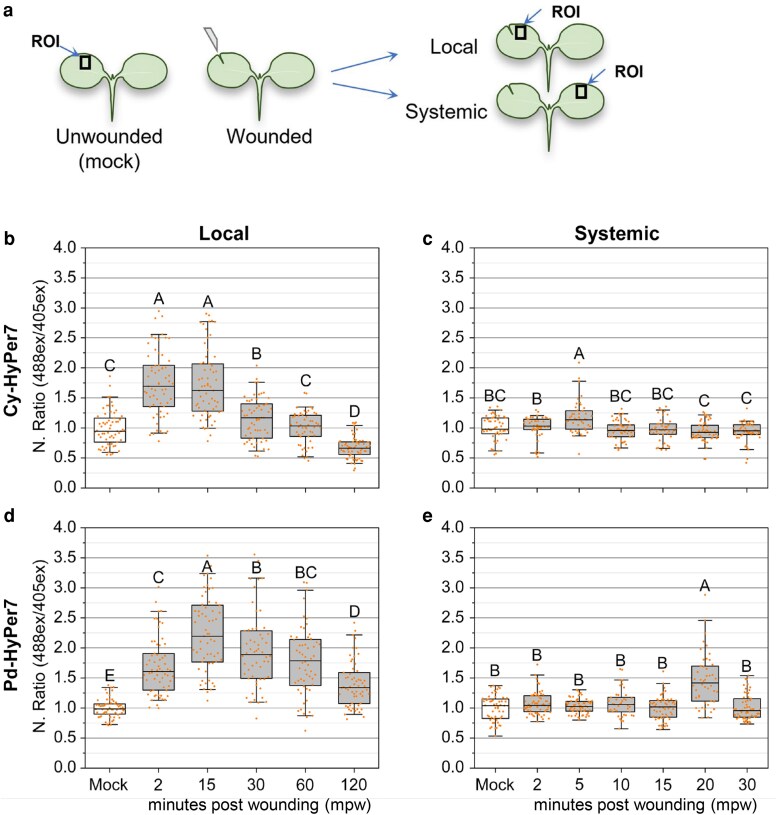
Systemic wound response reveals a distinct plasmodesmal redox behavior. (a) A scheme illustrating the experimental setup for recording wound-induced local and systemic H_2_O_2_ bursts. For local responses, one cotyledon per seedling was wounded by making a small incision, and the area adjacent to the wound site was imaged. For systemic responses, H_2_O_2_ dynamics were monitored in the opposite, unwounded cotyledon. (b) and (c) Box plots of Cy-HyPer7 ratios at the indicated times post-wounding in local (b) and systemic (c) cotyledons. *n* = 20 to 30 images from *n* > 12 to 15 seedlings per condition from two independent experiments. (d) and (e) Box plots of Pd-HyPer7 ratios at the indicated times post-wounding in local (d) and systemic (e) cotyledons. *n* = 40 to 50 images from *n* > 20 to 25 seedlings per condition from two independent experiments. Data are normalized to the mean ratio of mock-treated (unwounded) seedlings. Statistical analyses as in Fig. 1.

Previously, we have shown that wound-induced plasmodesmal restriction is transient (Cui and Lee 2016), but if wounding induces a systemic plasmodesmal response remains unknown. Having found a rapid H_2_O_2_ burst at plasmodesmata, we lastly examined the temporal aspects of wound-induced oxidative burst at plasmodesmata compared with that in the cytosol. Specifically, we asked how long the burst persists in local tissues and how this local event is signaled to systemic tissues. To address these questions, we wounded one cotyledon by making a small slit in half the cotyledon and measured Pd- and Cy-HyPer7 ratios in matched ROIs over a 2-hour time course in the wounded cotyledon (local) and for 30 min in the other cotyledon (systemic) (Fig. 9a). Upon wounding, both the cytosol and plasmodesmata exhibited rapid ROS bursts in wounded cotyledon, which was detected by the earliest time point (2 min post-wounding [mpw]) that reached maximal values within the early time window (2 to 15-mpw) (Fig. 9, b and d). Cy-HyPer7 subsequently declined toward mock level by 60-mpw and dropped below the mock level by 120-mpw (Fig. 9b). In systemic cotyledon, Cy-HyPer7 exhibited a modest transient increase at 5-mpw and returned toward its mock level by 10-mpw (Fig. 9c). In contrast, Pd-HyPer7 remained elevated at 60-mpw and decreased by 120-mpw, substantially above its mock level at this time point (Fig. 9d). Moreover, systemic Pd-HyPer7 responses were delayed, with a transient peak occurring at 20-mpw, which returned toward its mock level by 30-mpw (Fig. 9e). These data reveal distinct local versus systemic kinetics, and a temporal separation between early transient cytosolic oxidation and delayed plasmodesmal oxidation in systemic tissues, thereby defining the timing of systemic plasmodesmal oxidation relative to the systemic cytosolic oxidation burst.

### Discussion

Collectively, our results establish Pd-HyPer7 as a tool for monitoring redox dynamics at plasmodesmata in living plant tissues. Under defined exogenous H_2_O_2_ and DTT treatments, Pd-HyPer7 revealed redox response properties that differed from the same sensor targeted to other compartments. We note that these differences cannot represent differences in absolute H_2_O_2_ concentrations in each compartment. However, the rapid Pd-HyPer7 responses to exogenous treatments, the modulation of Pd-HyPer7 by targeted APX, and delayed systemic Pd-HyPer7 response compared to Cy-HyPer7 after wounding support the idea that despite lacking a complete membrane enclosure, plasmodesmata partake in spatially regulated redox signaling during stress responses.

In recent years, plasmodesmata have been increasingly recognized for their role in facilitating both local and/or systemic movement of various signaling molecules, including H_2_O_2_, calcium, and hormones (Lu et al. 2018; Toyota et al. 2018; Fichman et al. 2021, 2023; Peláez-Vico et al. 2022). These signaling molecules interestingly show a reciprocal relationship with plasmodesmata in that they regulate plasmodesmal permeability while their cell-to-cell movement depends on functional plasmodesmata (Sager et al. 2020; Wang et al. 2023). Among these signals, H_2_O_2_ has been directly linked to a rapid and transient plasmodesmal closure; and, intact plasmodesmal function has been shown to be required for a normal propagation of the H_2_O_2_ wave, which is crucial for plant acclimation to abiotic stresses (Rutschow et al. 2011; Cui and Lee 2016; Fichman et al. 2021). We have previously shown that the plasmodesmata-specific callose synthase, CalS8, is required for the H_2_O_2_-dependent plasmodesmal closure (Cui and Lee 2016). Based on the rapidness of this response, we reasoned that H_2_O_2_ likely acts locally to activate the enzymatic activity of CalS8, which points to the existence of localized H_2_O_2_ at plasmodesmata.

Our current study addressed this possibility by developing a HyPer7-based reporter that can monitor redox sensitivity and dynamics at plasmodesmata. Unlike the cytosol and PM, which show redox responses to exogenously applied H_2_O_2_ at the millimolar range, plasmodesmata become saturated at this range, exhibiting dose-dependent redox responses only at much lower, sub-millimolar concentrations. This unexpected, enhanced sensitivity to H_2_O_2_ and distinct response pattern suggests plasmodesmata may serve as early detection sites for oxidative signals at cell-cell boundaries. Moreover, the observed rapid and gradual redox responses at sub-millimolar H_2_O_2_ concentrations may define a regulatory or physiological range that is potentially crucial for signal processing at these junctions. These redox characteristics align with the established role of plasmodesmata in transmitting both local and systemic signals during stress responses and defense activation.

The sub-millimolar H_2_O_2_ measurements were made possible by the ultrasensitive, and ultrafast, HyPer7 sensor (Pak et al. 2020). HyPer7 was first adapted for plants to study H_2_O_2_ in the cytosol of root epidermal cells (Ugalde et al. 2021b), and similar to that study, we found that HyPer7 can detect lower, physiological levels of H_2_O_2_. In contrast, the overall redox state measured with Pd-roGFP2 was less sensitive to exogenous H_2_O_2_. Future studies could target roGFP2 fused to the oxidant receptor peroxidase-1 (Orp1) (Roma et al. 2018) or the faster TSA2ΔC_R_ peroxiredoxin (Morgan et al. 2016) to enhance specificity to H_2_O_2_, but in the cytosol of plants, these had a relative insensitivity to exogenously applied H_2_O_2_, which was attributed to a high buffering capacity of the cytosol due to the glutathione-based antioxidant system (Schwarzlander et al. 2008; Niemeyer et al. 2021; Ugalde et al. 2021a). Thus, we chose HyPer7 for this study and did not pursue roGFP2 fusion variants. However, the sensitivity and speed of HyPer7 also bring challenges. Prior use of HyPer7 in roots used standard laser scanning confocal microscopy (LSCM) (Ugalde et al. 2021b), but we found that in leaf tissue, LSCM was too slow for the ultrafast HyPer7 variant, which is 60 times faster than the original (Pak et al. 2020). HyPer7 responds to changes in H_2_O_2_ on the time scale of milliseconds rather than seconds, and thus, changes in chloroplastic H_2_O_2_ caused by the scanning laser could be detected within a single image. Consequently, for our study, a spinning disk confocal microscope was required to match the speed and sensitivity of HyPer7.

Another point of caution is that any axial chromatic aberrations between 405 and 488 nm excitation can alter the HyPer7 ratio calculations of sub-resolution structures, like plasmodesmata. This can be avoided by using a highly corrected objective lens or by taking the ratio of maximum intensity projections. However, a strict requirement for the HyPer sensors is that the 405 and 488 nm channels must be acquired sequentially, and thus, any focal drift or sample movement can alter the ratio values. Therefore, it was difficult to determine if the fast flickering or heterogeneity of individual Pd-HyPer7 spots we observed (Videos S3 and S6) was a technical artifact or real rapid responses of individual plasmodesmata. Fast spinning disk confocal microscopy can minimize the effect of this limitation, and we are exploring approaches that enable us to characterize individual plasmodesmal spots in follow-up studies. Lastly, a caveat of using the HyPer7 sensor is that the sensitivity is concentration dependent in in vitro assays (Pak et al. 2020). We were unable to show similar concentration dependence *in planta*, but we cannot fully eliminate the possibility that the local HyPer7 concentration and local environment of the sensor are affecting the measurements. Therefore, the tool should not be used to calculate absolute concentrations of H_2_O_2,_ and comparisons between compartments should be conducted with this caveat considered. Despite these challenges and limitations, the speed and sensitivity of HyPer7 made it possible to not only detect sub-millimolar H_2_O_2_, but also to examine the dynamics of H_2_O_2_ responses with time-lapse imaging.

Given that low amounts of exogenous H_2_O_2_ rapidly saturate plasmodesmata, we speculate that they likely function as redox sensors rather than primary sites for H_2_O_2_ detoxification. The precise, local H_2_O_2_ concentration at plasmodesmata from these treatments is unknown, primarily due to uncertainties in how much H_2_O_2_ reaches plasmodesmata and the potential for the interplay of other cellular responses in altering H_2_O_2_ levels at plasmodesmata. However, the observed low buffering capacity and responses at physiological, sub-millimolar concentrations are consistent with a sensory role that coordinates H_2_O_2_ responses across cellular boundaries. This saturation of redox responses at higher H_2_O_2_ concentrations may represent a protective mechanism, limiting the excessive spread of ROS or the passage of damaging molecules. Such a protective role would be crucial as the early ROS detection would enable cells to mount a timely and effective response to oxidative stress, thereby preventing damage and maintaining cellular homeostasis both locally and systemically.

The role of these rapid redox responses on plasmodesmal function remains to be determined experimentally. However, it is tantalizing to speculate that the distinct redox responses we observed below and above the saturation point might enable complex, differential regulation of plasmodesmal function. This possibility is supported by a previous study reporting an interesting biphasic plasmodesmal regulation, where a low concentration (0.6 mM) increased permeability while a high concentration (6 mM) decreased it (Rutschow et al. 2011). Although direct comparison is not possible due to different experimental conditions, we speculate that the stepwise increase in H_2_O_2_-induced oxidation could translate into differential regulation of plasmodesmal function, such as permeability, protein trafficking, or downstream signaling. Alternatively, the graded response to H_2_O_2_ levels could act as a distribution control system, facilitating H_2_O_2_ movement at low concentrations while restricting the spread of excessive oxidative stress at higher levels, ultimately enabling plants to fine-tune their responses to varying levels of oxidative signals.

SA and ROS are crucial signaling molecules in plant defense and abiotic stress signaling, contributing to local and systemic acquired immunity and stress tolerance (Mateo et al. 2006; Kachroo and Kachroo 2020; Devireddy et al. 2021; Spoel and Dong 2024). While some studies suggest they function in parallel (El-Shetehy et al. 2015), the precise functional relationship between SA and ROS remains complex. Our systematic analysis of *NahG* plants has revealed several key insights into plasmodesmal redox regulation and its relationship with SA signaling. Notably, the SA-deficient *NahG* line maintained normal H_2_O_2_ response patterns at plasmodesmata, despite altered redox responses in other compartments. However, the underlying redox capacity of these plants appears distinct. Previous studies indicate that depending on environmental conditions, SA-deficiency reconditions thiol buffering capacity and homeostasis rather than simply lowering basal H_2_O_2_ levels. For example, SA-deficient *Arabidopsis NahG* seedlings maintain a more reduced glutathione pool (higher GSH/GSSG ratio) compared to WT under salt and osmotic stress conditions (Borsani et al. 2001) and both *NahG* and *sid2* mutant plants are predisposed to oxidative stress under high light stress (Mateo et al. 2006). Our data further provide evidence for a compartment-specific shift in redox status in mock-treated *NahG* plants.

While *NahG* chloroplasts were significantly more reduced than WT under mock treatment, *NahG* plasmodesmata maintained a redox state statistically indistinguishable from WT. This suggests that plasmodesmata possess local homeostatic mechanisms that actively maintain a specific redox setpoint even in an SA-deficient background. However, SA deficiency may push the buffering system at plasmodesmata toward its limit such that additional reductants paradoxically trigger oxidative responses. Importantly, exogenous SA did not alter redox states at plasmodesmata in WT plants, consistent with our conclusion that plasmodesmata have compartment-specific redox regulatory mechanisms that operate independently of SA signaling. Thus, while plasmodesmal redox regulation appears independent of acute SA signaling, persistent SA deficiency likely reshapes the cellular redox homeostasis, increasing the demand on local buffering at plasmodesmata to maintain its setpoint. We propose that maintaining this setpoint likely places *NahG* plasmodesmata under “reductive stress,” operating near their buffering limit to counteract the systemic reductive load.

Consequently, when challenged with additional reducing power (DTT), this fragile equilibrium collapses, leading *NahG* plants to exhibit a paradoxical oxidative response to DTT in both the cytosol and plasmodesmata. This parallels our result that DTT treatment increased oxidation in WT plants expressing Pd-APX. In both cases, the addition of a reductant triggered an oxidative burst, a hallmark of reductive stress described in human cells as an imbalance in redox couples [such as NAD(H) or GSH(GSSG)] that leads to paradoxical ROS generation (Xiao and Loscalzo 2020). Disruption of these shuttles can cause localized reductive stress in one compartment while triggering oxidative stress in another, making reductive stress equally detrimental to organismal function as oxidative stress. Reductive stress responses have also been reported in plants, where ER has been shown to be sensitive to reductive stress and mitochondrial oxidation safeguards ER via retrograde signaling (Fuchs et al. 2022; Ugalde et al. 2022). Our findings extend this framework to plasmodesmata, suggesting that they may represent another subcellular domain where reductive limits are tightly governed to prevent destabilization of the local signaling environment.

Our real-time monitoring revealed stress-dependent redox responses reported by Cy- and Pd-HyPer7. During cold treatment, both reporters showed broadly similar oxidation dynamics, which suggest plasmodesmata are not universally faster or stronger in responses than the cytosol. In contrast, mechanical wounding revealed distinct local and systemic response profiles. Notably, an early transient H_2_O_2_ burst was detected in the cytosol. A transient burst was also detected at plasmodesmata but with a delay. This temporal separation provides evidence that plasmodesmata function as a distinct redox compartment. In addition, the gradual oxidation observed at plasmodesmata even under gentle water flow suggests a mechanosensitive redox property that warrants further investigation. These findings are consistent with plasmodesmata acting not merely as passive targets but as sites where environmental perturbations are transduced into localized redox changes at intercellular junctions. Given that H_2_O_2_ can modulate plasmodesmal permeability, these data also support a model in which plasmodesmata participate in redox-based stress sensing at cell-cell interfaces.

Rapid systemic signaling in plants can involve propagating ROS and calcium signals, but the route of signal propagation and the role of plasmodesmata remain debated. In the context of systemic acclimation to high light, Fichman et al. (2021) showed that systemic ROS signaling requires RBOHD-generated ROS and involves plasmodesmal regulators for propagation of systemic ROS signals, which move at velocities of several centimeters per minute (Fichman et al. 2021). In the context of mechanical wounding, however, Bellandi et al. (2022) proposed an alternative framework in which wound-induced vascular calcium waves can be explained by apoplastic diffusion and bulk flow of amino acids acting as mobile chemical messengers moving at comparable speeds. They also reported that wound-induced calcium wave dynamics were not altered in *rbohd/rbohf* backgrounds nor impeded by inducing plasmodesmal closure through callose accumulation (Bellandi et al. 2022). Thus, both models predict a rapid arrival of the systemic signal within minutes, but differ in whether plasmodesmata actively propagate or passively receive the signal. Our cotyledon-to-cotyledon time-course experiments do not directly identify the primary long-distance messenger; however, they resolve the timing of oxidative dynamics within the cytosol and plasmodesmata and their relationship to plasmodesmal regulation.

Two observations (Fig. 9) support a model in which plasmodesmata function as sensors or integrators of wound signals rather than as conduits for ROS propagation: First, in local epidermal cells the cytosolic oxidation response resolves rapidly and falls below mock level by 120-mpw, whereas plasmodesmal H_2_O_2_ levels remain elevated longer, indicating distinct kinetics between the cytosol and plasmodesmata. Second, systemic responses are temporally separated, with the cytosolic transient burst peaking at 5-mpw and the plasmodesmal burst at ∼20-mpw. This lag or uncoupling implies that the plasmodesmal response is not a simple reflection of bulk cytosolic oxidation and likely involves additional regulatory steps and/or thresholding at plasmodesmata. Importantly, the delayed systemic plasmodesmal peak (∼20 mpw) provides a testable timing prediction that any systemic plasmodesmal permeability response (e.g. callose-dependent restriction) would be expected to occur after the early cytosolic burst, aligning with this later plasmodesmal oxidation window. Regardless, the temporal uncoupling is more consistent with plasmodesmata responding as downstream targets of earlier systemic signals (Bellandi et al. 2022). In this view, the distinct redox sensitivity of plasmodesmata positions them not as early warning relays, but as decision points that coordinate intercellular connectivity with the magnitude and duration of stress. This interpretation is also consistent with plasmodesmata being membrane-rich structures that are proposed to be enriched in receptor-like functions, signaling capacity, as well as redox-associated proteins (Fernandez-Calvino et al. 2011). Indeed, to enable a targeted ROS response apart from bulk cytosolic influence, it is reasonable to hypothesize a dedicated redox regulatory machinery associated with plasmodesmata. Candidate components include RBOHD-linked ROS production at plasmodesmata, plasmodesmata-associated peroxidases, and local thiol redox regulators that could set a threshold for plasmodesmal oxidation. The tools and timing framework established in our current study (including the delayed systemic plasmodesmal oxidation window) provide direct, testable predictions for dissecting these mechanisms genetically and pharmacologically. Future work will be required to define the upstream signals and molecular components that couple systemic wound perception to delayed plasmodesmal oxidation.

In summary, our findings using HyPer7 sensors offer new perspectives on redox responses at plasmodesmata. The dynamic responses reported by Pd-HyPer7 and the temporally uncoupled systemic response during wounding support a model in which plasmodesmata participate in spatially and temporally regulated redox signaling across cellular boundaries. Moving forward, defining the plasmodesmal redox regulators that set the redox sensitivity and dynamics will be essential to uncover the underlying molecular mechanisms.

## Methods

### Plant materials and growth conditions

N. *benthamiana* WT and *NahG* transgenic plants were grown in a controlled environment under diurnal conditions of 18 h light (120 to 180 μmol m^−2^ s^−1^) at 23 °C and 6 h dark at 21 °C with 60% relative humidity for 24 h.

Flowering *Arabidopsis* Col-0 plants were transformed with the same binary vector used for transient expression in *N. benthamiana.* Independent transgenic lines were screened using confocal microscopy to identify lines with suitable expression levels in target compartments and sensor functionality. A minimum of 10 to 20 lines were screened for each of Cy- and Ch-HyPer7, while over 50 lines were screened for Pd-HyPer7. Specific transgenic lines used for ratio imaging were: Cy-HyPer7 (T2-4), Ch-HyPer7 (T2-3), and Pd-HyPer7 (T2-27). Selected T2 or T3 Arabidopsis seedlings were grown vertically on ½ Murashige Skoog (MS) agar plates under ∼80 to 100 µmol m^−2^ s^−1^ light intensity with a 16 h/8 h light/dark cycle. For flow system experiments and wounding, seedlings were grown on MS agar under the growth chamber conditions of ∼50 µmol m^−2^ s^−1^ light intensity with a 10 h/14 h light/dark cycle and 65% humidity. Seven-day-old seedlings were used for all treatments and ratio imaging experiments.

### Plasmid cloning

Recombinant DNA constructs were produced by overlapping PCR using high-fidelity Q5 Taq Polymerase (New England Biolabs [NEB]), followed by subcloning of gel-purified PCR fragments into *Sfi*I-digested pMB binary vector (Sager et al. 2020) using T4 DNA ligase (NEB). All other enzymes, including restriction enzymes, were supplied by NEB. The HyPer7 sequence was amplified using an Addgene plasmid (pCS2 + HyPer7) as the template. The APX sequence (AtAPX1, At1g07890) was amplified from cDNA synthesized from total RNA isolated from *Arabidopsis* leaves. All plasmid clones expressing fusions of two proteins carry a glycine linker (RPGGGGGP) between them. The clone expressing a fusion of three proteins (PDL5-APX-mKate2) contains the glycine linker between PDL5-APX and an alanine linker (PAGAAAAAAGA) between APX and mKate2. The DNA sequence of each PCR-cloned plasmid construct was confirmed for its fidelity using Sanger or whole plasmid sequencing. All DNA primers used in this study are provided in Table S1.

To produce PDLP5 fusion to roGFP2, HyPer7, mKate2, or APX, the full-length ORF of each gene was PCR amplified with gene-specific primers containing *Age*I and *Xba*I sites at the 5′- and 3′ ends, respectively, and subcloned into pMB35S:*Sfi*I-PDLP5-EGFP (Wang et al. 2020) plasmid by replacing the *EGFP* fragment. The mTM-HyPer7 or -mKate2 was produced via subcloning *Sfi*I-digested *mTM* sequence into pMB35S:*SfiI*-PDLP5-HyPer7/mKate2 by replacing *PDLP5*. The *mTM* DNA fragment was digested from the pMB35S-based vector containing a PM-localized PDLP5 variant (Luna et al. 2023). To produce chloroplast-localized constructs, the DNA region encoding the first 79-amino acid residues of the RbcS was PCR amplified from *N. benthamiana* leaf cDNA using 5′- and 3′-primers containing a unique *Sfi*I site at each end. *Sfi*I-digested PCR fragment was subsequently subcloned into pMB35S:SfiI-PDLP5-HyPer7, -mKate2, or APX-mKate2, replacing the *PDLP5* sequence. To produce cytosol-localized constructs, HyPer7 or mKate2 was PCR amplified, using 5′-primer containing an *Sfi*I site and 3′-primer containing another *Sfi*I site followed by the DNA sequence encoding an NES (LPPLERLTL) (see Table S1 for primer sequence information) adopted from the original cytosolic HyPer7-NES (Pak et al. 2020). *Sfi*I-digested PCR amplicon was subcloned into the vector pMB35S:*Sfi*I, which contains two *Sfi*I sites that allow for a directional cloning of the insert. APX-mKate2 was produced by overlapping PCR using 5′- and 3′-end primers that contain directional *Sfi*I sites, followed by cloning into pMB35S*Sfi*I. To produce PM-localized constructs, the EGFP in pMB35S:PM-GFP was replaced with HyPer7 or mKate2 using *Age*I and *Xba*I. pMB35S:PM-GFP consists of a PM-localized PDLP5 variant resulting from a swapping mutation in its JMe and TMD domains with the corresponding segments derived from BAK1, a PM-localizing membrane protein (Luna et al. 2023).

### *Agrobacterium*-mediated transient expression in *N. benthamiana*

*Agrobacterium tumefaciens* cells of strain GV3101 (+pSoup) were transformed with each pMB plasmid harboring a specific DNA construct by electroporation. Transformants were selected by culturing the electroporation competent cells overnight at 28 °C, on LB-agar medium supplemented with antibiotics (gentamicin 50 μg/ml, rifampicin 50 μg/ml, tetracycline 10 μg/ml, and spectinomycin 200 μg/ml). For a liquid culture, a single positive colony was picked and grown overnight at 28 °C by shaking at 225 rpm, in LB medium containing the same antibiotics. For agroinfiltration, cells were harvested and resuspended in an infiltration buffer (10 mM MES, pH 5.7, 10 mM MgSO_4_, and 100 µM acetosyringone [PhtoTech LABS, A104]) to an optical density of 0.1 to 0.3 at 600 nm. The resuspended *Agrobacterium* cells were syringe-infiltrated into mature leaves of 3- to 4-week-old *N. benthamiana* plants, which were kept in a growth chamber for an additional 4 to 5 d until they were imaged by confocal microscopy.

### Chemical and mechanical wounding treatments

Chemicals (DTT [bioWORLD, #40400120 bioPLUS] and H_2_O_2_ [Fisher Scientific, # 7722-84-1]) used to treat plants were prepared in water freshly each time immediately before use to the desired concentrations. Water was used for mock treatment. Leaves of *N. benthamiana* were infiltrated with the prepared solutions using gentle syringe infiltration without a needle. Immediately following infiltration, small sections of treated leaves were excised and mounted in water or chemical treatments using single-well Lab-TekII Chambers with cover glass bottoms (Thermo Scientific, #155360) for imaging. Mechanical wounding treatment was performed using 7-day-old *Arabidopsis* seedlings by making a small incision on one cotyledon with a fine razor blade, extending less than halfway across its width to avoid severing the midvein, followed by confocal imaging of either the wounded (local) or unwounded (systemic) cotyledon. For local tissue, epidermal cells were imaged several cell layers away from the visible wound site to ensure recording of intact cells adjacent to the wound site.

### Confocal microscopy for subcellular localization

Spinning disk confocal microscopy images were acquired on an Andor Dragonfly 600 (Oxford Instruments, Belfast, UK) with a Leica HC Plan Apochromat CS2 40 × water immersion objective lens [numerical aperture, 1.10] and a Zyla Plus 4.2 CMOS camera. The 405 and 488 nm laser lines were used to excite the 400 and 500 nm absorption peaks of HyPer7 or roGFP2 and fluorescence was collected with a 512/38 nm emission filter. The images acquired using the 405 nm excitation (405ex) and 488 nm excitation (488ex) were acquired sequentially and used for 488ex/405ex ratiometric calculations and image display. mKate2 and mCherry were excited with the 561 nm laser and chlorophyll autofluorescence with the 638 nm laser. The µW of laser power at the objective, percentage laser power, and exposure time can be found in Table S2.

### Flow chamber system

An FCS2 flow chamber (Bioptechs Inc., Butler, PA) was modified for real-time monitoring of redox dynamics in intact *Arabidopsis* seedlings (Figure S19). The chamber was assembled using adhesive-backed Secure-Seal spacers (Thermofisher, #S24737), which remain adherent in water, to hold the seedling. Seven-day-old seedlings were carefully positioned in the chamber with roots oriented away from the curved edges to prevent damage, and the assembly was sealed to create a flow-through system. The flow chamber was connected to a 60 mL syringe equipped with a stopcock via 15 cm of tubing to control solution delivery. The mounted seedling was positioned on a microscope stage for continuous imaging of cotyledon epidermal cells while treatment solutions flowed through the chamber. For experimental treatments, baseline recordings were collected for 2 min before introducing test solutions (H_2_O_2_ at various concentrations, ice-cold water at 2 °C, or mock treatments with room temperature water). Flow rate was controlled by gravity (6.8 ml/min), with the syringe system allowing for rapid switching between different treatment solutions during time-course experiments. The system enabled continuous monitoring of cellular responses throughout the treatment period, typically over 20-minute time courses. A single focal plane was imaged to maximize the speed of the image acquisition.

### Ratio image data processing

Ratio image processing was conducted using batch processing in ImageJ FIJI (Schindelin et al. 2012). In brief, raw spinning disk confocal microscopy z-stacks (2 µm thick) were first flattened using a maximum intensity z-projection. Noise was reduced with Gaussian Blur filtering, and the camera background level was adjusted. A 488ex/405ex or 405ex/488ex ratio image was created for HyPer7 and roGFP2, respectively. Analysis masks were created by thresholding and were used to measure the ratios at subcellular locations. The Analyze Particles function in ImageJ was used to calculate the maximum ratio value for each PD and the mean ratio value for chloroplasts, PM, or cytosolic regions. For each field of view, ratio values were averaged across all measurable structures for plasmodesmal puncta and chloroplasts, and cytosol or PM regions across multiple cells. This yielded a single average ratio value per image. For time-lapse datasets, an average of mock treatment values from 3- to 4-mpt was used to normalize ratios (N. ratio) to 1.0 to aid in the comparison of datasets. For single time point datasets, an average of all mock treatment replicates was used as the normalization value for the ratios. For flow chamber single focal plane datasets, an average of mock treatment values from −1 to 0 min (pretreatment) was first used to normalize the ratio, and then ratios were adjusted so that the ratio was exactly 1.0 at 0 min for all treatments. A complete workflow and more specifications can be found in Figure S2. Representative image time series were selected and drift-corrected in Huygens Professional Version 25.04. The stabilized data were then converted to an uncompressed AVI at 10 frames per second using Fiji. The resulting video files were arranged using KapWing software.

The OxD dose-response data were fitted with the Hill equation using the following formula: OxD = START + (END − START) × [H_2_O_2_]^n^/(EC_50_^n^ + [H_2_O_2_]^n^), where START was fixed at the mock baseline OxD for each compartment, END was fixed at 1.0, EC_50_ represents the half-maximal effective concentration, and n is the Hill coefficient. Curve fitting was performed using Origin 2024 software with instrumental weighting (1/SEM^2^). *R*^2^ values are reported in Fig. 3g.

### Statistical analysis

Statistical analyses were performed using R or Origin. Kruskal–Wallis tests were used to assess overall differences among treatment groups, followed by Conover's post-hoc tests with Benjamini–Hochberg correction for pairwise comparisons. Two-way ANOVA followed by Tukey's post-hoc test for pairwise comparisons was used to evaluate interaction effects between two different treatments or between treatment and genotype. Significance was set at *α* = 0.05. Ordinal logistic regression (proportional odds model) was used for categorized GFP movement outcomes (Figure S10).

## Supplementary Material

koag192_Supplementary_Data

## Data Availability

Data supporting the findings of this study are available in the manuscript and its Supplementary files or are available from the corresponding authors upon request. The source data underlying Figs 1, f and i, 2c, 3, g and h, 4, b, d, and f to h, 5, c to k, 6, b, d, and g, and 9, b to e; and Figures S4, S7, and S9 to S15 are provided as the Source Data file.

## References

[koag192-B1] Asada K . 2006. Production and scavenging of reactive oxygen species in chloroplasts and their functions. Plant Physiol. 141:391–396. 10.1104/pp.106.082040. 16760493 PMC1475469

[koag192-B2] Bayer EM, Benitez-Alfonso Y. 2024. Plasmodesmata: channels under pressure. Annu Rev Plant Biol. 75:291–317. 10.1146/annurev-arplant-070623-093110. 38424063

[koag192-B3] Bellandi A et al 2022. Diffusion and bulk flow of amino acids mediate calcium waves in plants. Sci Adv. 8:eabo6693. 10.1126/sciadv.abo6693. PMC9586480 36269836

[koag192-B4] Bilska A, Sowinski P. 2010. Closure of plasmodesmata in maize (*Zea mays*) at low temperature: a new mechanism for inhibition of photosynthesis. Ann Bot. 106:675–686. 10.1093/aob/mcq169. 20880933 PMC2958785

[koag192-B5] Booth DM, Varnai P, Joseph SK, Hajnoczky G. 2021. Oxidative bursts of single mitochondria mediate retrograde signaling toward the ER. Mol Cell. 81:3866–3876.e2. 10.1016/j.molcel.2021.07.014. 34352204 PMC8455442

[koag192-B6] Borsani O, Valpuesta V, Botella MA. 2001. Evidence for a role of salicylic acid in the oxidative damage generated by NaCl and osmotic stress in Arabidopsis seedlings. Plant Physiol. 126:1024–1030. 10.1104/pp.126.3.1024. 11457953 PMC116459

[koag192-B7] Cheval C et al 2020. Chitin perception in plasmodesmata characterizes submembrane immune-signaling specificity in plants. Proc Natl Acad Sci U S A. 117:9621–9629. 10.1073/pnas.1907799117. 32284410 PMC7196898

[koag192-B8] Cui W, Lee J-Y. 2016. Arabidopsis callose synthases CalS1/8 regulate plasmodesmal permeability during stress. Nat Plants. 2:16034. 10.1038/nplants.2016.34. 27243643

[koag192-B9] Devireddy AR, Zandalinas SI, Fichman Y, Mittler R. 2021. Integration of reactive oxygen species and hormone signaling during abiotic stress. Plant J. 105:459–476. 10.1111/tpj.15010. 33015917

[koag192-B10] Dopp IJ, Kalac K, Mackenzie SA. 2023. Hydrogen peroxide sensor HyPer7 illuminates tissue-specific plastid redox dynamics. Plant Physiol. 193:217–228. 10.1093/plphys/kiad307. 37226328 PMC10702466

[koag192-B11] Ehlers K, van Bel AJE. 2010. Dynamics of plasmodesmal connectivity in successive interfaces of the cambial zone. Planta. 231:371–385. 10.1007/s00425-009-1046-8. 19936780

[koag192-B12] El-Shetehy M et al 2015. Nitric oxide and reactive oxygen species are required for systemic acquired resistance in plants. Plant Signal Behav. 10:e998544. 10.1080/15592324.2014.998544. 26375184 PMC4883869

[koag192-B13] Exposito-Rodriguez M, Laissue PP, Yvon-Durocher G, Smirnoff N, Mullineaux PM. 2017. Photosynthesis-dependent H_2_O_2_ transfer from chloroplasts to nuclei provides a high-light signalling mechanism. Nat Commun. 8:49. 10.1038/s41467-017-00074-w. 28663550 PMC5491514

[koag192-B14] Faulkner C et al 2013. LYM2-dependent chitin perception limits molecular flux via plasmodesmata. Proc Natl Acad Sci U S A. 110:9166–9170. 10.1073/pnas.1203458110. 23674687 PMC3670346

[koag192-B15] Fernandez-Calvino L et al 2011. Arabidopsis plasmodesmal proteome. PLoS One. 6:e18880. 10.1371/journal.pone.0018880. 21533090 PMC3080382

[koag192-B16] Fichman Y, Myers RJ Jr, Grant DG, Mittler R. 2021. Plasmodesmata-localized proteins and ROS orchestrate light-induced rapid systemic signaling in *Arabidopsis*. Sci Signal. 14:eabf0322. 10.1126/scisignal.abf0322. 33622982

[koag192-B17] Fichman Y, Rowland L, Oliver MJ, Mittler R. 2023. ROS are evolutionary conserved cell-to-cell stress signals. Proc Natl Acad Sci U S A. 120:e2305496120. 10.1073/pnas.2305496120. 37494396 PMC10400990

[koag192-B18] Foyer CH, Kunert K. 2024. The ascorbate-glutathione cycle coming of age. J Exp Bot. 75:2682–2699. 10.1093/jxb/erae023. 38243395 PMC11066808

[koag192-B19] Foyer CH, Noctor G. 2011. Ascorbate and glutathione: the heart of the redox hub. Plant Physiol. 155:2–18. 10.1104/pp.110.167569. 21205630 PMC3075780

[koag192-B20] Friedrich L, Vernooij B, Gaffney T, Morse A, Ryals J. 1995. Characterization of tobacco plants expressing a bacterial salicylate hydroxylase gene. Plant Mol Biol. 29:959–968. 10.1007/BF00014969. 8555459

[koag192-B21] Fuchs P et al 2022. Reductive stress triggers ANAC017-mediated retrograde signaling to safeguard the endoplasmic reticulum by boosting mitochondrial respiratory capacity. Plant Cell. 34:1375–1395. 10.1093/plcell/koac017. 35078237 PMC9125394

[koag192-B22] Gousset K, Marzo L, Commere P-H, Zurzolo C. 2013. Myo10 is a key regulator of TNT formation in neuronal cells. J Cell Sci. 126:4424–4435. 10.1242/jcs.129239. 23886947

[koag192-B23] Huang H et al 2025. Activation of a FOXO3-induced cell cycle arrest regulates ferroptosis. Cell Death Discov. 11:465. 10.1038/s41420-025-02760-x. 41102177 PMC12533257

[koag192-B24] Kachroo A, Kachroo P. 2020. Mobile signals in systemic acquired resistance. Curr Opin Plant Biol. 58:41–47. 10.1016/j.pbi.2020.10.004. 33202317

[koag192-B25] Ledo A, Fernandes E, Salvador A, Laranjinha J, Barbosa RM. 2022. In vivo hydrogen peroxide diffusivity in brain tissue supports volume signaling activity. Redox Biol. 50:102250. 10.1016/j.redox.2022.102250. 35101799 PMC8804256

[koag192-B26] Lee J-Y et al 2011. A plasmodesmata-localized protein mediates crosstalk between cell-to-cell communication and innate immunity in Arabidopsis. Plant Cell. 23:3353–3373. 10.1105/tpc.111.087742. 21934146 PMC3203451

[koag192-B27] Li Z, Liu S-L, Montes-Serey C, Walley JW, Aung K. 2024. PLASMODESMATA-LOCATED PROTEIN 6 regulates plasmodesmal function in Arabidopsis vasculature. Plant Cell. 36:3543–3561. 10.1093/plcell/koae166. 38842334 PMC11371196

[koag192-B28] Lim G-H et al 2016. Plasmodesmata localizing proteins regulate transport and signaling during systemic acquired immunity in plants. Cell Host Microbe. 19:541–549. 10.1016/j.chom.2016.03.006. 27078071

[koag192-B29] Liu T-L et al 2018. Observing the cell in its native state: imaging subcellular dynamics in multicellular organisms. Science. 360:eaaq1392. 10.1126/science.aaq1392. 29674564 PMC6040645

[koag192-B30] Lu K-J, Danila FR, Cho Y, Faulkner C. 2018. Peeking at a plant through the holes in the wall—exploring the roles of plasmodesmata. New Phytol. 218:1310–1314. 10.1111/nph.15130. 29574753

[koag192-B31] Luna GR, Li J, Wang X, Liao L, Lee J-Y. 2023. Targeting of plasmodesmal proteins requires unconventional signals. Plant Cell. 35:3035–3052. 10.1093/plcell/koad152. 37225403 PMC10396362

[koag192-B32] Mateo A et al 2006. Controlled levels of salicylic acid are required for optimal photosynthesis and redox homeostasis. J Exp Bot. 57:1795–1807. 10.1093/jxb/erj196. 16698814

[koag192-B33] Mignolet-Spruyt L et al 2016. Spreading the news: subcellular and organellar reactive oxygen species production and signalling. J Exp Bot. 67:3831–3844. 10.1093/jxb/erw080. 26976816

[koag192-B34] Miller EW, Dickinson BC, Chang CJ. 2010. Aquaporin-3 mediates hydrogen peroxide uptake to regulate downstream intracellular signaling. Proc Natl Acad Sci U S A. 107:15681–15686. 10.1073/pnas.1005776107. 20724658 PMC2936599

[koag192-B35] Mittler R et al 2011. ROS signaling: the new wave? Trends Plant Sci. 16:300–309. 10.1016/j.tplants.2011.03.007. 21482172

[koag192-B36] Morgan B, Sobotta MC, Dick TP. 2011. Measuring E(GSH) and H2O2 with roGFP2-based redox probes. Free Radic Biol Med. 51:1943–1951. 10.1016/j.freeradbiomed.2011.08.035. 21964034

[koag192-B37] Morgan B et al 2016. Real-time monitoring of basal H2O2 levels with peroxiredoxin-based probes. Nat Chem Biol. 12:437–443. 10.1038/nchembio.2067. 27089028

[koag192-B38] Murata Y, Nagata K, Abe M. 2025. Cell-to-cell translocation of florigen is inhibited by low ambient temperature through abscisic acid signaling in *Arabidopsis thaliana*. P Natl Acad Sci U S A. 122:e2507987122. 10.1073/pnas.2507987122. PMC12280980 40623194

[koag192-B39] Nedo AO et al 2024. CHUP1 restricts chloroplast movement and effector-triggered immunity in epidermal cells. New Phytol. 244:1864–1881. 10.1111/nph.20147. 39415611 PMC11583462

[koag192-B40] Niemeyer J, Scheuring D, Oestreicher J, Morgan B, Schroda M. 2021. Real-time monitoring of subcellular H2O2 distribution in Chlamydomonas reinhardtii. Plant Cell. 33:2935–2949. 10.1093/plcell/koab176. 34196712 PMC8462822

[koag192-B41] Pak VV et al 2020. Ultrasensitive genetically encoded indicator for hydrogen peroxide identifies roles for the oxidant in cell migration and mitochondrial function. Cell Metab. 31:642–653.e6. 10.1016/j.cmet.2020.02.003. 32130885 PMC7088435

[koag192-B42] Peláez-Vico MA et al 2022. ROS and redox regulation of cell-to-cell and systemic signaling in plants during stress. Free Radic Biol Med. 193:354–362. 10.1016/j.freeradbiomed.2022.10.305. 36279971

[koag192-B43] Petrov VD, Van Breusegem F. 2012. Hydrogen peroxide-a central hub for information flow in plant cells. AoB Plants. 2012:pls014. 10.1093/aobpla/pls014. 22708052 PMC3366437

[koag192-B44] Podgorska A, Burian M, Szal B. 2017. Extra-cellular but extra-ordinarily important for cells: apoplastic reactive oxygen Species metabolism. Front Plant Sci. 8:1353. 10.3389/fpls.2017.01353. 28878783 PMC5572287

[koag192-B45] Roma LP, Deponte M, Riemer J, Morgan B. 2018. Mechanisms and applications of redox-sensitive green fluorescent protein-based hydrogen peroxide probes. Antioxid Redox Signal. 29:552–568. 10.1089/ars.2017.7449. 29160083

[koag192-B46] Rustom A . 2016. The missing link: does tunnelling nanotube-based supercellularity provide a new understanding of chronic and lifestyle diseases? Open Biol. 6:160057. 10.1098/rsob.160057. 27278648 PMC4929939

[koag192-B47] Rutschow HL, Baskin TI, Kramer EM. 2011. Regulation of solute flux through plasmodesmata in the root meristem. Plant Physiol. 155:1817–1826. 10.1104/pp.110.168187. 21325566 PMC3091107

[koag192-B48] Sager R, Lee J-Y. 2014. Plasmodesmata in integrated cell signalling: insights from development and environmental signals and stresses. J Exp Bot. 65:6337–6358. 10.1093/jxb/eru365. 25262225 PMC4303807

[koag192-B49] Sager R et al 2020. Auxin-dependent control of a plasmodesmal regulator creates a negative feedback loop modulating lateral root emergence. Nat Commun. 11:364. 10.1038/s41467-019-14226-7. 31953391 PMC6969147

[koag192-B50] Schindelin J et al 2012. Fiji: an open-source platform for biological-image analysis. Nat Methods. 9:676–682. 10.1038/nmeth.2019. 22743772 PMC3855844

[koag192-B51] Schwarzlander M et al 2008. Confocal imaging of glutathione redox potential in living plant cells. J Microsc. 231:299–316. 10.1111/j.1365-2818.2008.02030.x. 18778428

[koag192-B52] Shcherbo D et al 2009. Far-red fluorescent tags for protein imaging in living tissues. Biochem J. 418:567–574. 10.1042/BJ20081949. 19143658 PMC2893397

[koag192-B53] Sies H . 2017. Hydrogen peroxide as a central redox signaling molecule in physiological oxidative stress: oxidative eustress. Redox Biol. 11:613–619. 10.1016/j.redox.2016.12.035. 28110218 PMC5256672

[koag192-B54] Smirnoff N, Arnaud D. 2019. Hydrogen peroxide metabolism and functions in plants. New Phytol. 221:1197–1214. 10.1111/nph.15488. 30222198

[koag192-B55] Spoel SH, Dong XN. 2024. Salicylic acid in plant immunity and beyond. Plant Cell. 36:1451–1464. 10.1093/plcell/koad329. 38163634 PMC11062473

[koag192-B56] Stonebloom S et al 2012. Redox states of plastids and mitochondria differentially regulate intercellular transport via plasmodesmata. Plant Physiol. 158:190–199. 10.1104/pp.111.186130. 22074709 PMC3252087

[koag192-B57] Tee EE, Faulkner C. 2024. Plasmodesmata and intercellular molecular traffic control. New Phytol. 243:32–47. 10.1111/nph.19666. 38494438

[koag192-B58] Tee EE, Johnston MG, Papp D, Faulkner C. 2023. A PDLP-NHL3 complex integrates plasmodesmal immune signaling cascades. Proc Natl Acad Sci U S A. 120:e2216397120. 10.1073/pnas.2216397120. 37068237 PMC10151459

[koag192-B59] Toyota M et al 2018. Glutamate triggers long-distance, calcium-based plant defense signaling. Science. 361:1112–1115. 10.1126/science.aat7744. 30213912

[koag192-B60] Ugalde JM et al 2021a. Chloroplast-derived photo-oxidative stress causes changes in H2O2 and EGSH in other subcellular compartments. Plant Physiol. 186:125–141. 10.1093/plphys/kiaa095. 33793922 PMC8154069

[koag192-B61] Ugalde JM et al 2022. Endoplasmic reticulum oxidoreductin provides resilience against reductive stress and hypoxic conditions by mediating luminal redox dynamics. Plant Cell. 34:4007–4027. 10.1093/plcell/koac202. 35818121 PMC9516139

[koag192-B62] Ugalde JM, Schlosser M, Dongois A, Martiniere A, Meyer AJ. 2021b. The latest HyPe(r) in plant H2O2 biosensing. Plant Physiol. 187:480–484. 10.1093/plphys/kiab306. 34608965 PMC8491017

[koag192-B63] Veal E, Day A. 2011. Hydrogen peroxide as a signaling molecule. Antioxid Redox Signal. 15:147–151. 10.1089/ars.2011.3968. 21375475

[koag192-B64] Vu MH et al 2022. ROS-mediated plasmodesmal regulation requires a network of an Arabidopsis receptor-like kinase, calmodulin-like proteins, and callose synthases. Front Plant Sci. 13:1107224. 10.3389/fpls.2022.1107224. 36743578 PMC9893415

[koag192-B65] Wang X, Luna GR, Arighi CN, Lee J-Y. 2020. An evolutionarily conserved motif is required for plasmodesmata-located protein 5 to regulate cell-to-cell movement. Commun Biol. 3:291. 10.1038/s42003-020-1007-0. 32504045 PMC7275062

[koag192-B66] Wang X et al 2013. Salicylic acid regulates plasmodesmata closure during innate immune responses in Arabidopsis. Plant Cell. 25:2315–2329. 10.1105/tpc.113.110676. 23749844 PMC3723628

[koag192-B67] Wang Y, Cui J, Sun X, Zhang Y. 2011. Tunneling-nanotube development in astrocytes depends on p53 activation. Cell Death Differ. 18:732–742. 10.1038/cdd.2010.147. 21113142 PMC3131904

[koag192-B68] Wang Y et al 2023. Plasmodesmata mediate cell-to-cell transport of brassinosteroid hormones. Nat Chem Biol. 19:1331–1341. 10.1038/s41589-023-01346-x. 37365405 PMC10729306

[koag192-B69] Waszczak C, Carmody M, Kangasjarvi J. 2018. Reactive oxygen Species in plant signaling. Annu Rev Plant Biol. 69:209–236. 10.1146/annurev-arplant-042817-040322. 29489394

[koag192-B70] Xiao W, Loscalzo J. 2020. Metabolic responses to reductive stress. Antioxid Redox Signal. 32:1330–1347. 10.1089/ars.2019.7803. 31218894 PMC7247050

[koag192-B71] Young M et al 2024. Transcriptional regulation in the absence of inositol trisphosphate receptor calcium signaling. Front Cell Dev Biol. 12:1473210. 10.3389/fcell.2024.1473210. 39712573 PMC11659226

[koag192-B72] Zanini AA, Burch-Smith TM. 2024. New insights into plasmodesmata: complex ‘protoplasmic connecting threads’. J Exp Bot. 75:5557–5567. 10.1093/jxb/erae307. 39001658 PMC11427835

