# Supplementary material for: Plasmodesmata display dynamic local and systemic redox responses during plant stress
Source: Plant Cell. 2026 Jun 22;38(7):koag192. doi: 10.1093/plcell/koag192 (PMC13412043; doi:10.1093/plcell/koag192)
Supplement: koag192_Supplementary_Data [file koag192_supplementary_data.zip › Supplemental Figures.pdf]

Supplementary Figure 1

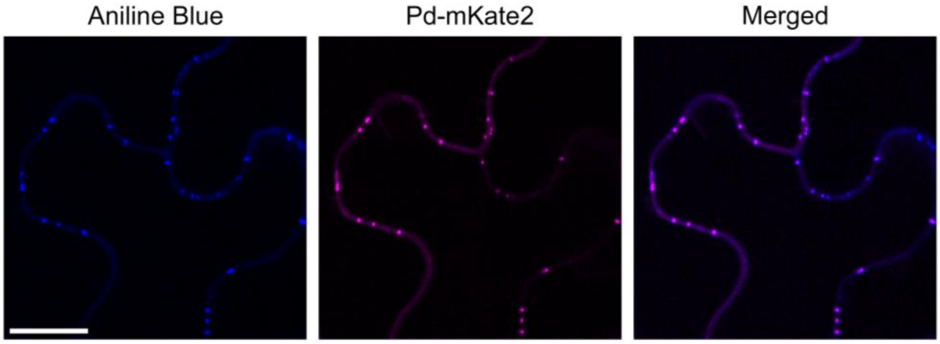

Plasmodesmal localization of Pd-mKate2. *Agrobacterium* cells (GV3101) carrying a binary vector expressing Pd-mKate2 (pMB35S:PDLP5-mKate2) were infiltrated into the leaves of 3.5-week-old *N. benthamiana* plants. Four to five days post-infiltration, leaves were infiltrated with 0.02% aniline blue and abaxial leaf epidermal cells were imaged by confocal microscopy to assess co-localization with callose. Experiments were repeated at least three times. Scale bar, 20 μm. This figure supplements Figure 1B results.

Supplementary figure 2

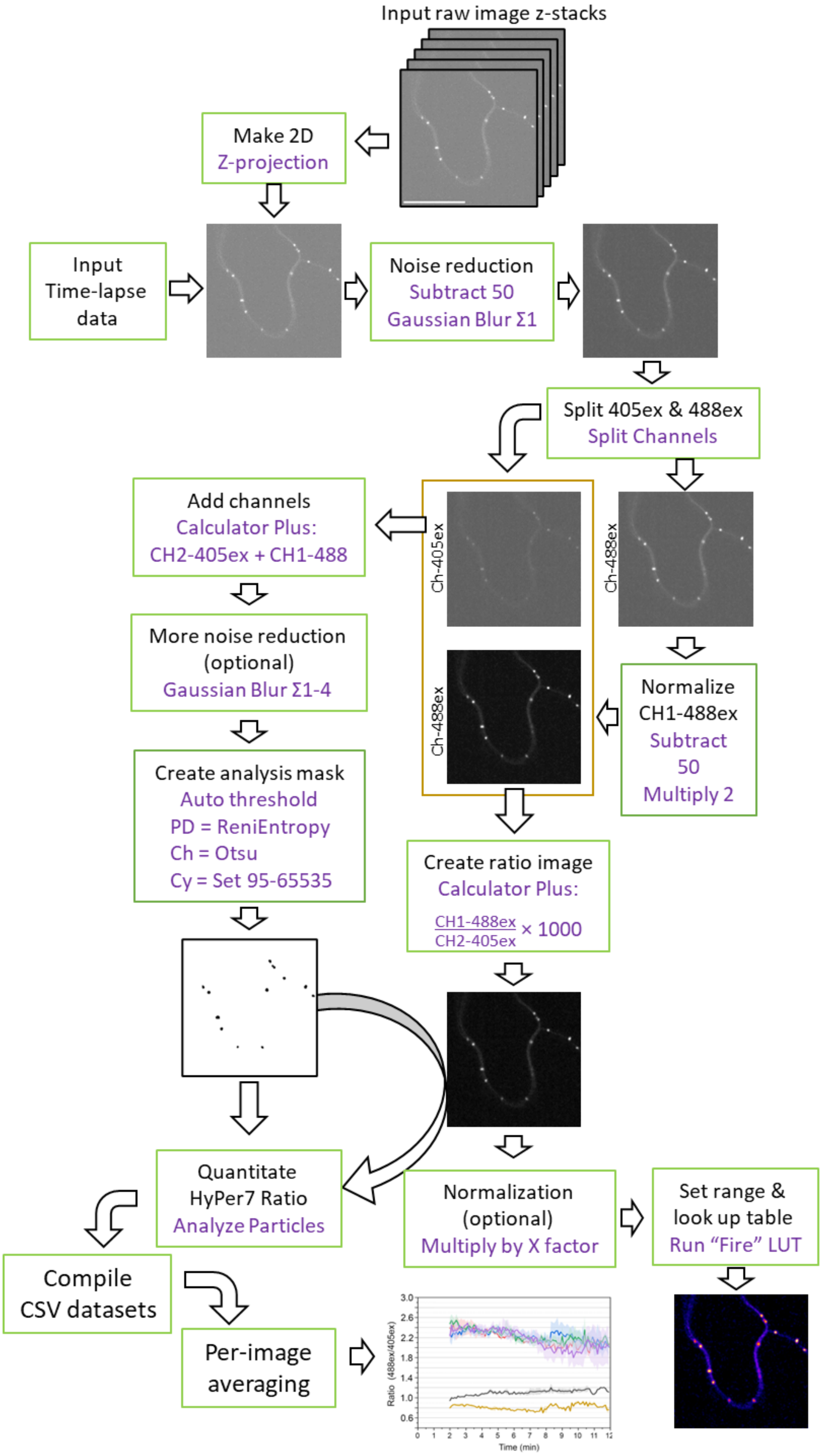

Supplementary figure 2, continued

Workflow illustrating the step-by-step process for acquisition and processing ratiometric images. (1) Abaxial leaf epidermal cells of *N. benthamiana* transiently expressing the sensor (or transgenic *Arabidopsis* in later experiments) are imaged, immediately following treatments. Ratiometric imaging is performed using a spinning disk confocal microscope (Andor DragonFly), capturing separate images at 488 nm and 405 nm excitation wavelengths. (2) The confocal images are subsequently processed to automatically capture fluorescent signals using custom ImageJ macro scripts, which perform background subtraction, thresholding, and ratio calculation. For quantitative analyses, the mean ratio of all auto-detected spots within each image was used as a single data point. The ratio intensity values are then normalized for comparative quantification analyses. (3) The visualization of ratio images involves applying a ratio-based lookup table (LUT) to create the pseudo-color visualization of redox states across the imaged area, where the color intensity represents the 488 nm/405 nm ratio, indicative of the cellular redox state. This standardized workflow ensures consistent and unbiased quantitative analysis of redox status in leaf epidermal cells across all experimental conditions. This figure supplements Figure 1C results.

Supplementary Figure 3

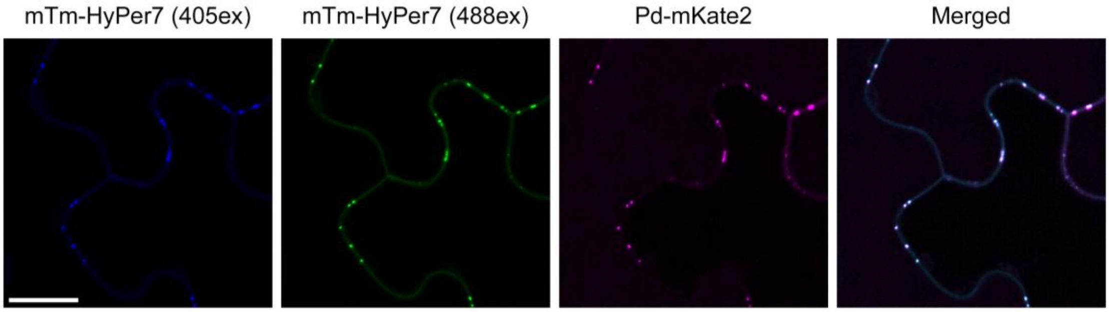

mTm-HyPer7 localizes to plasmodesmata. A representative set of confocal images showing co-localization of mTM-HyPer7 with Pd-mKate2. *Agrobacterium* strains (GV3101) each transformed with pMB35S binary vector expressing mTm-HyPer7 or Pd-mKate2 were cultured and co-infiltrated into the leaves of 3.5-week-old *N. benthamiana* plants. Four to five days post-infiltration, abaxial leaf epidermal cells were imaged by confocal microscopy to assess their co-localization at plasmodesmata. Experiments were repeated at least three times. Scale bar, 20 μm.

Supplementary figure 4

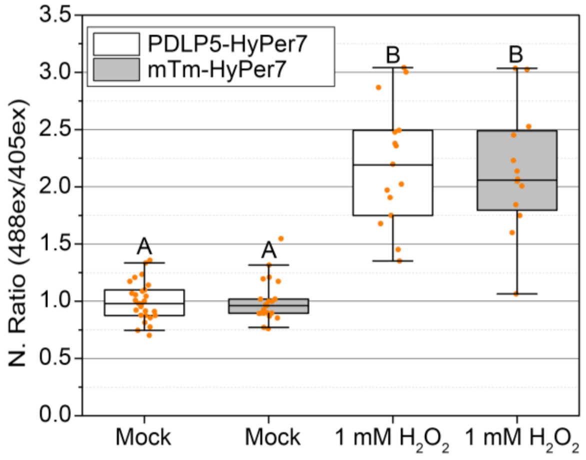

Quantitative analysis of ratiometric image data for PDLP5- and mTm-HyPer7 sensors transiently expressed in *N. benthamiana* leaf epidermal cells. A box plot showing the distribution of fluorescence intensity ratios (488 nm ex./405 nm ex.) for leaves treated with water (Mock) or 1 mM H_2O_2. Each box represents data from n = 15-25 images collected from 3 plants and two independent experiments. The line indicates the median and boxes the interquartile range, with whiskers extending to 1.5 times the interquartile range beyond the box boundaries. Individual data points are overlaid. Statistical analysis was performed using Kruskal-Wallis tests followed by Conover's post-hoc tests with Benjamini-Hochberg correction for pairwise comparisons ($\alpha = 0.05$). Different letters indicate statistically significant differences between groups with $p < 0.0001$.

Supplementary figure 5

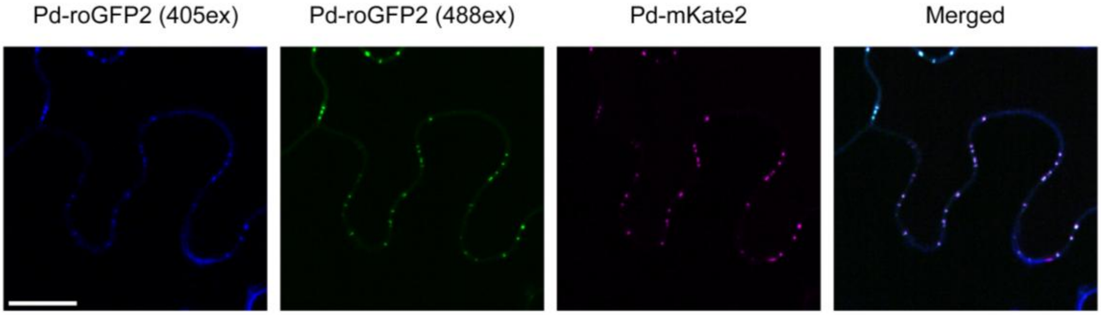

Plasmodesmal localization of Pd-roGFP2. *Agrobacterium* strains (GV3101), each carrying a binary vector expressing Pd-mKate2 or Pd-roGFP2 were cultured and co-infiltrated into the leaves of 3.5-week-old *N. benthamiana* plants. Four to five days post-infiltration, abaxial leaf epidermal cells were imaged by confocal microscopy to assess plasmodesmal localization of Pd-roGFP2. Experiments were repeated at least three times. Scale bar, 20 μm. This figure supplements Figure 1G results.

Supplementary figure 6

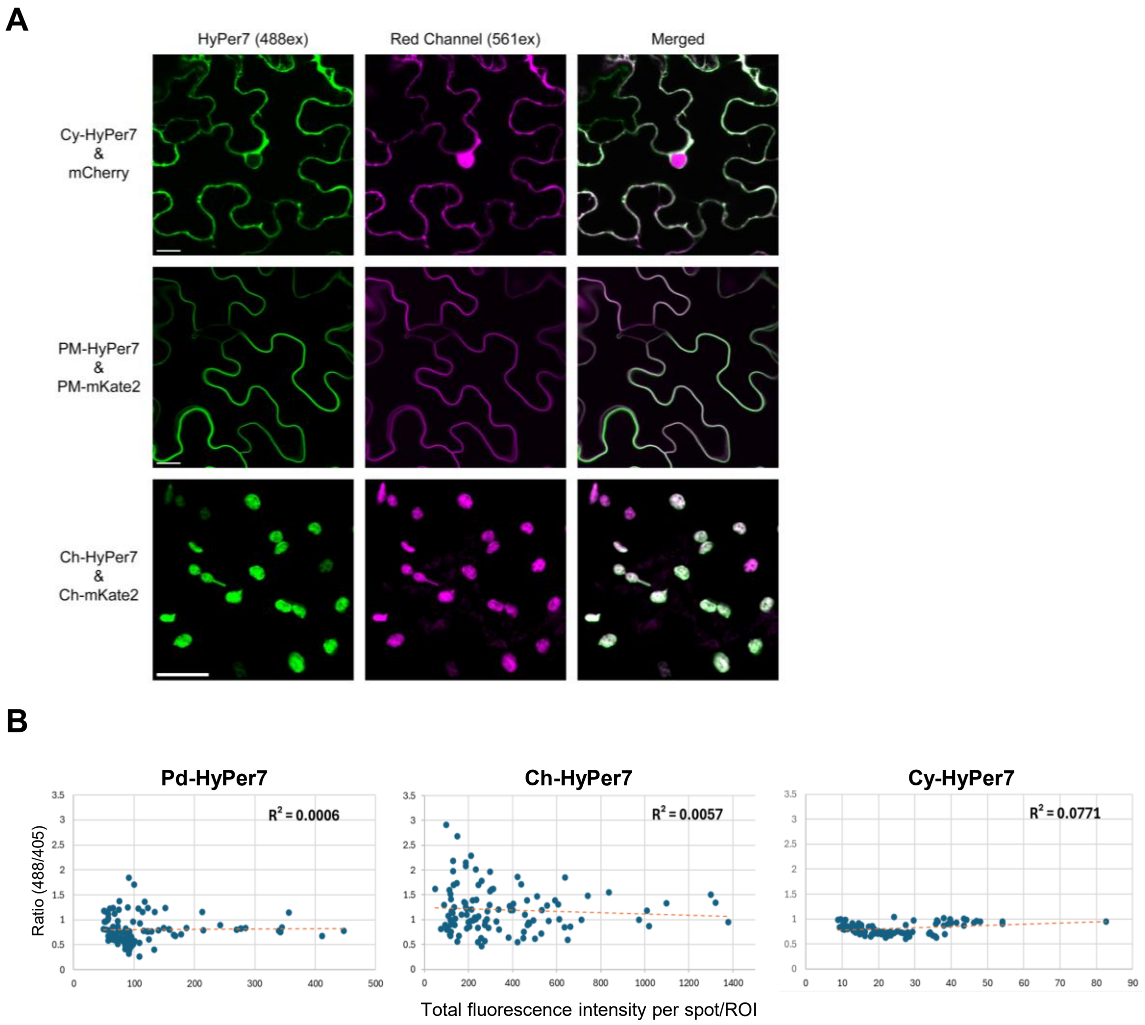

Compartmentally targeted HyPer7. A. Subcellular localization of HyPer7 targeted to cytosol, PM or chloroplasts. *Agrobacterium* cells (GV3101) carrying a binary vector expressing either mCherry or subcellularly targeted mKate2, along with each HyPer7 sensor, were co-infiltrated into the leaves of 3.5-week-old *N. benthamiana* plants. Four to five days post-infiltration, abaxial leaf epidermal cells were imaged by confocal microscopy. Scale bars, 20 μm. **B.** Scatter plots comparing HyPer7 488ex/405ex ratios with local total fluorescence intensity under mock conditions with linear regression analysis. X-axis, total HyPer7 fluorescence intensity per spot/ROI; Y-axis, corresponding 488ex/405ex HyPer7 ratio. Linear fits were used to test whether basal ratios showed a simple dependence on local reporter fluorescence intensity. The low R^2 values indicate no systematic relationship between local fluorescence intensity and ratio under mock conditions. This figure supplements Figure 3A, 3C, 3E and 3G results.

Supplementary figure 7

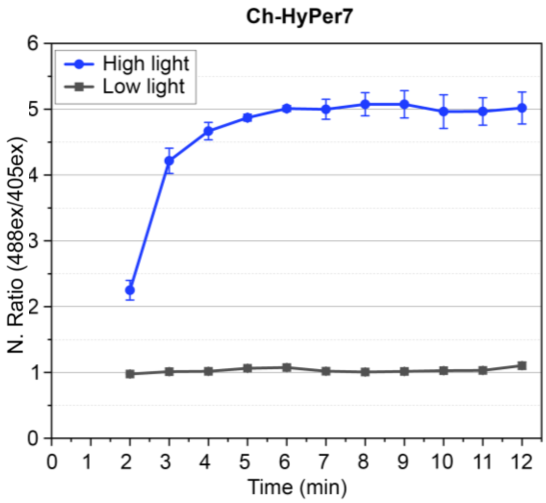

Time-lapse response of Ch-HyPer7 exposed to low or high light. The graph shows the ratiometric response (488 nm ex./405 nm ex.) of Ch-HyPer7 over 10-min under low (grey) and high (blue) laser light conditions (detailed settings in Table S1). The low laser power setting showed minimal change in ratio over time while the high light exposure rapidly increased the sensor ratio. Error bars represent standard error of the mean (n = 4 images).

Supplementary figure 8

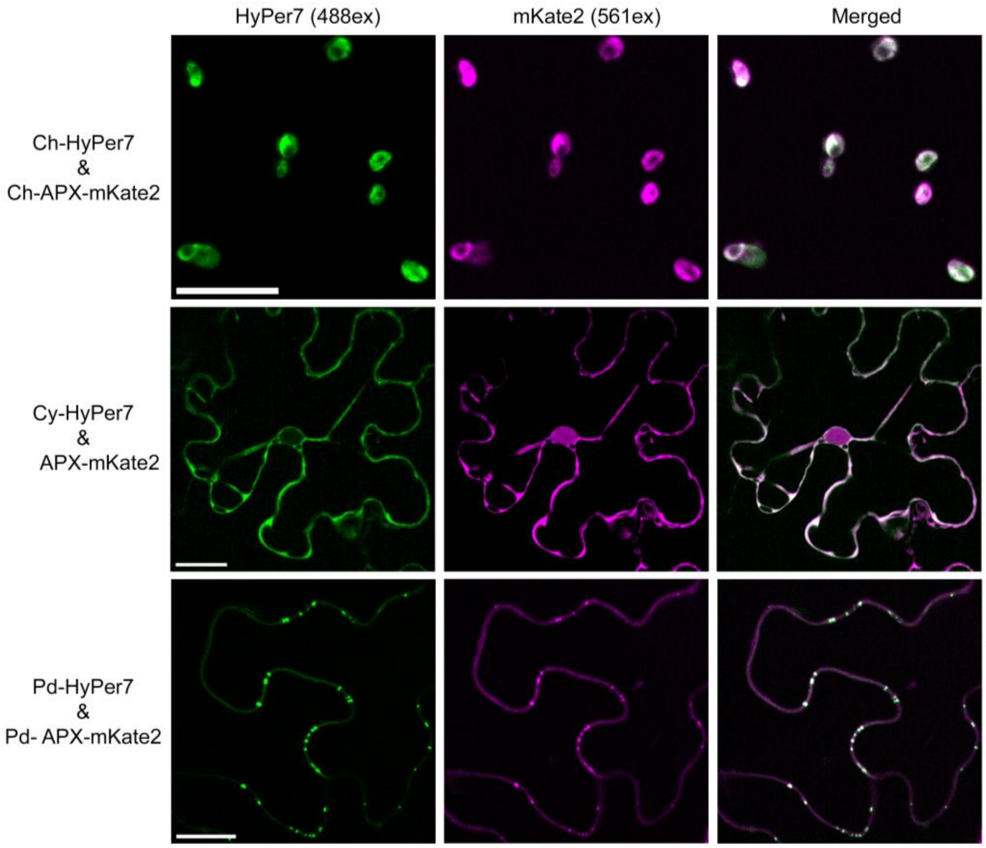

Representative confocal images showing APX targeted to chloroplasts, cytosol, and plasmodesmata. Correct targeting of each APX-mKate2 fusion proteins was verified by co-expression with respective HyPer7 sensors. To this end, *Agrobacterium* cells were co-infiltrated into the leaves of 3.5-week-old *N. benthamiana* plants. Four to five days post-infiltration, abaxial leaf epidermal cells were imaged by confocal microscopy. Scale bars, 20 µm. This figure supplements Figure 4A, 4C, and 4E results.

Supplementary figure 9

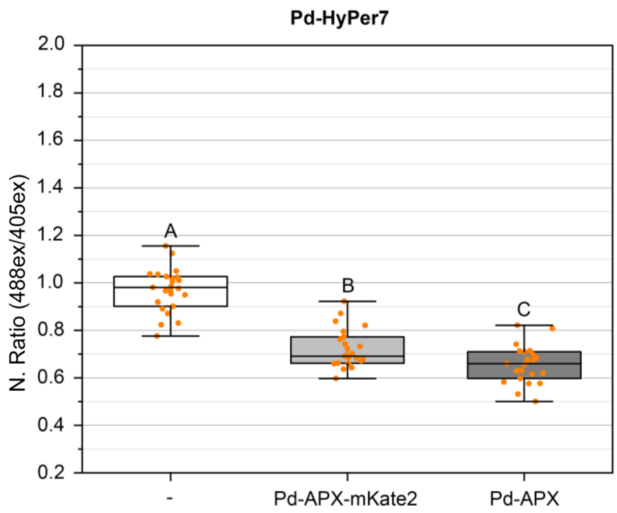

Pd-APX or Pd-APX-mKate2 are both similarly effective in reducing Pd-HyPer7. Graph shows quantitative analyses of fluorescence intensity ratios of Pd-HyPer7 in the absence (-) or presence of Pd-APX or Pd-APX-mKate2. Each box represents data from a total of n = 25 images using at least 6 plants from two independent experiments. Error bars indicate SE. Statistical analysis was performed using Kruskal-Wallis tests followed by Conover's post-hoc tests with Benjamini-Hochberg correction for pairwise comparisons ($\alpha = 0.05$). Different letters indicate statistically significant differences between groups with $p < 0.0001$. This figure supplements Figure 4E and 4F results.

Supplementary figure 10

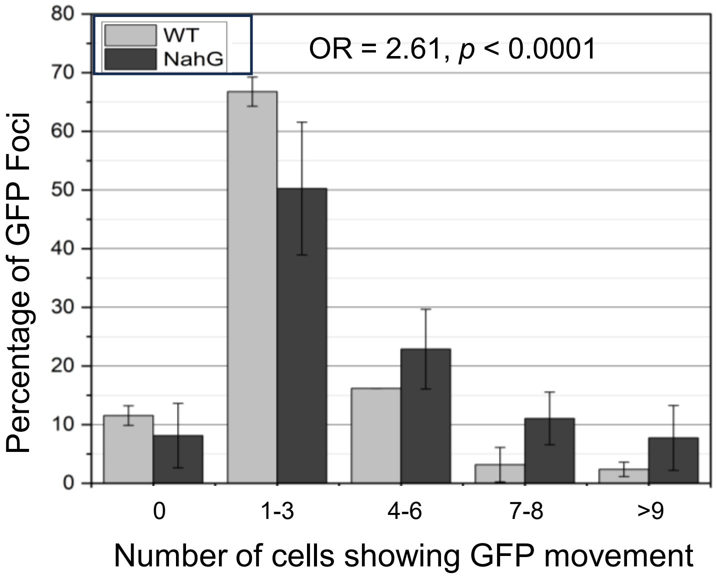

GFP movement assays showing an increased plasmodesmal permeability in *N. benthamiana NahG* plants. *Agrobacterium* cells carrying a binary vector expressing GFP were infiltrated into leaves of 3.5-week-old *N. benthamiana* WT and *NahG* plants. Three days post-infiltration, abaxial leaf epidermal cells were examined under a fluorescent microscope and scored for GFP movement. The experiment was replicated twice, using at least three plants per genotype per replicate. The extent of cell-to-cell movement is categorized into five different groups based on the number of cells showing GFP fluorescence. No movement of GFP from the transfected cell is scored as “0” cell. Statistical analysis using ordinal logistic regression (proportional odds model) revealed that *NahG* plants have significantly higher odds of more extensive GFP movement compared to WT (odds ratio = 2.61, 95% CI: 2.19–3.12, $p < 0.0001$; n = 996 WT foci and 984 *NahG* foci from two independent experiments). Error bars, SE. This figure supplements Figure 5 results.

Supplementary figure 11

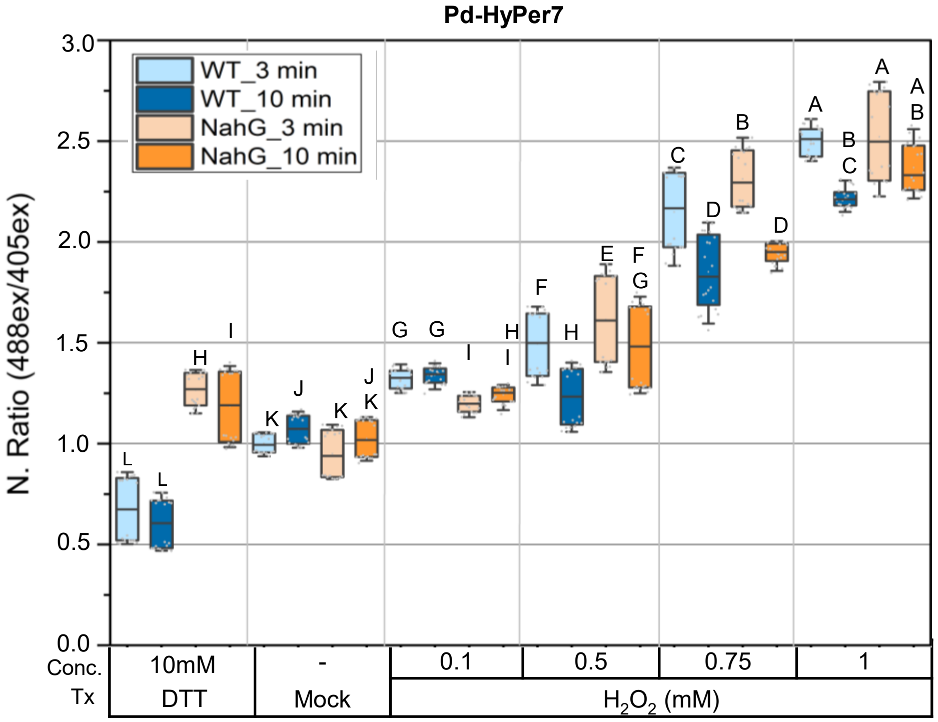

Time-lapse responses of Pd-HyPer7 in *NahG* to various redox treatments. Time-lapse changes of fluorescence intensity ratios of Pd-HyPer7 in *NahG* under different redox conditions. The WT data shown in the main Figure 2B were reused here. Data were collected a total of n = 6 images using at least four plants per treatment from two independent experiments. This experiment has been repeated three times and represented data from two independent experiments. Statistical analysis was performed using Kruskal-Wallis tests followed by Conover's post-hoc tests with Benjamini-Hochberg correction for pairwise comparisons (α = 0.05). Different letters indicate statistically significant differences between groups with $p < 0.0001$. This figure supplements Figures 2B and 5B results.

Supplementary figure 12

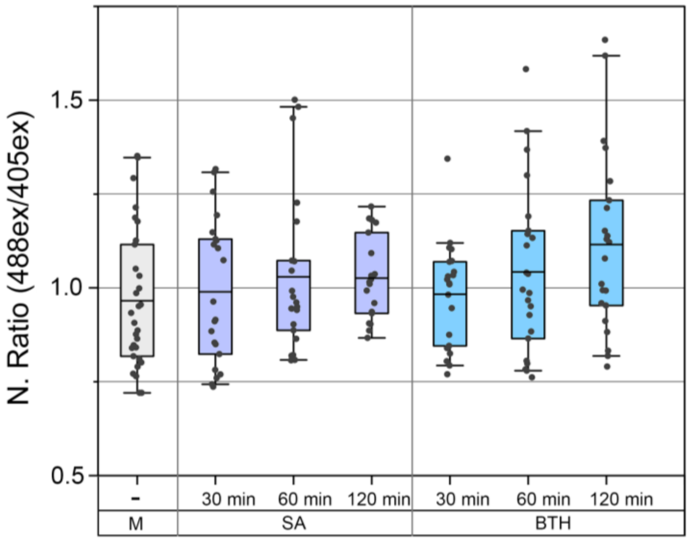

Quantitative analysis showing plasmodesmal redox levels are insensitive to exogenous SA treatments. WT *N. benthamiana* plants were treated with 100 µM SA (Sigma #S5922) or 100 µM BTH (S-methyl benzo[1,2,3]thiadiazole-7-carbothioate, Bion®, Syngenta) for 30 min, 1 hr and 2 hr before ratio imaging. Each treatment represents n = 15-25 images collected from 3 plants per repeat from two independent experiments. Statistical analysis performed using Kruskal-Wallis tests followed by Conover's post-hoc tests with Benjamini-Hochberg correction for pairwise comparisons ($\alpha = 0.05$) shows no statistical difference between Mock and SA/BTH treatments. This figure supplements Figure 5 results.

Supplementary figure 13

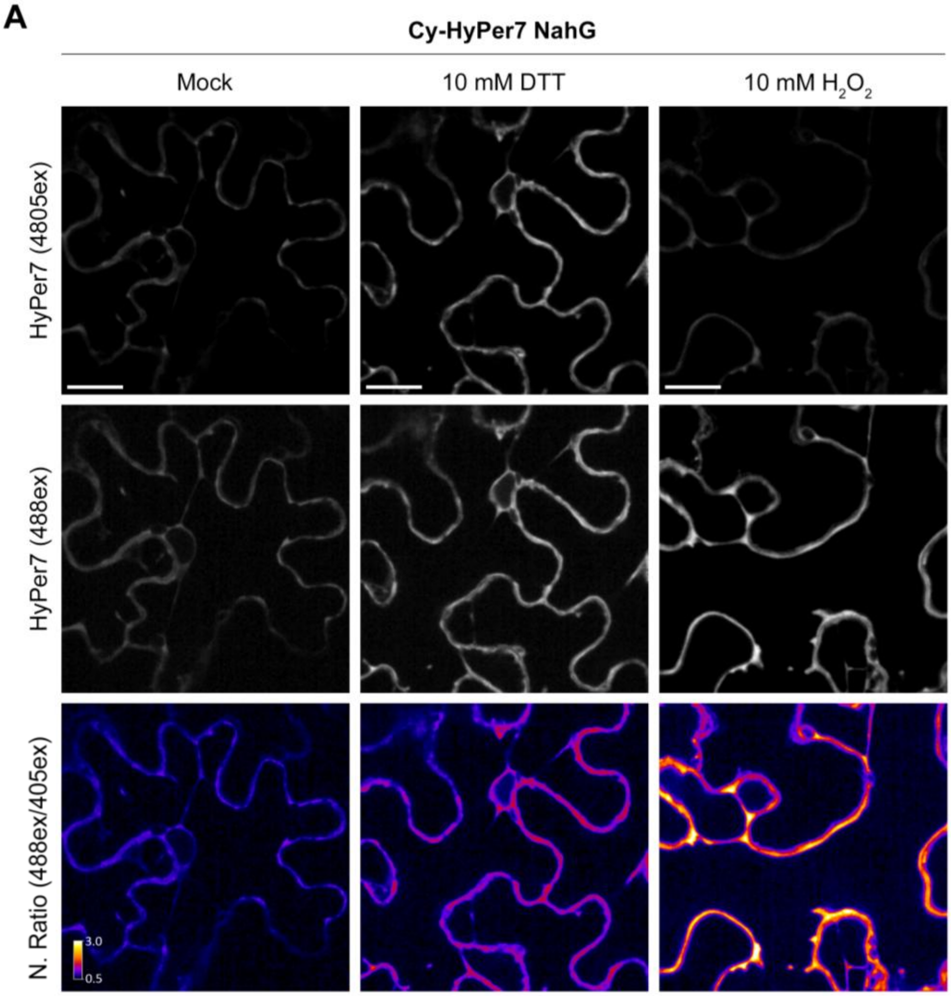

Supplementary figure 13, continued

B

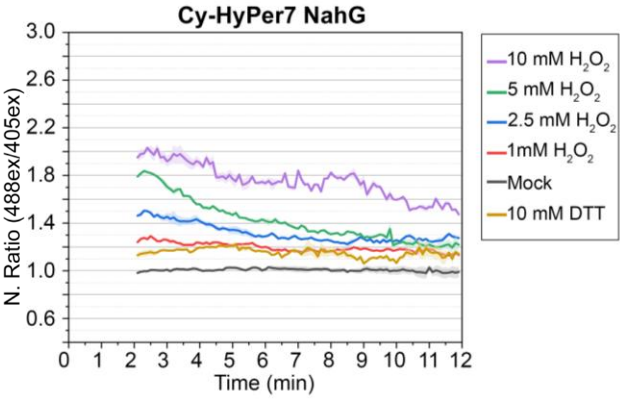

C

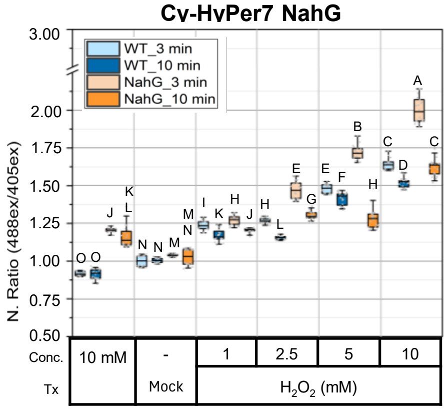

Time-lapse responses of Cy-HyPer7 in *NahG* to various redox treatments. (A) Representative fluorescence images of Cy-HyPer7 in *NahG*. Top panel, 405 nm excitation; middle panel, 488 nm excitation; and bottom panel, ratio image (488 nm ex./405 nm ex.). Images show responses to Mock, 10 mM DTT, and 10 mM H_2O_2 treatments at 3-4-mpt. Ratio values are represented by pseudocolor scale from 0.5 to 3.0. Scale bars, 20 µm. (B) Timelapse changes of fluorescence intensity ratios of Cy-HyPer7 in *NahG* under different redox conditions. Data were collected a total of n = 6 images using at least four plants per treatment from two independent experiments. This experiment has been repeated three times and represented data from two independent experiments. (C) Box plot showing relative fluorescence ratios (488 nm ex./405 nm ex.) of Cy-HyPer7 in WT and *NahG* leaf epidermal cells at early (3-4 min) and late (10-11 min) time points after each treatment. The ratios were normalized to those of mean ratios at 3-mpt in each experiment. Data was extracted from time-lapse data presented in B. Data were collected from a total of n = 6 images using at least four plants per treatment from two independent experiments. Statistical analysis was performed using Kruskal-Wallis tests followed by Conover's post-hoc tests with Benjamini-Hochberg correction for pairwise comparisons (α = 0.05). Different letters indicate statistically significant differences between groups (p < 0.0001). This figure supplements Figure 5 results.

Supplementary figure 14

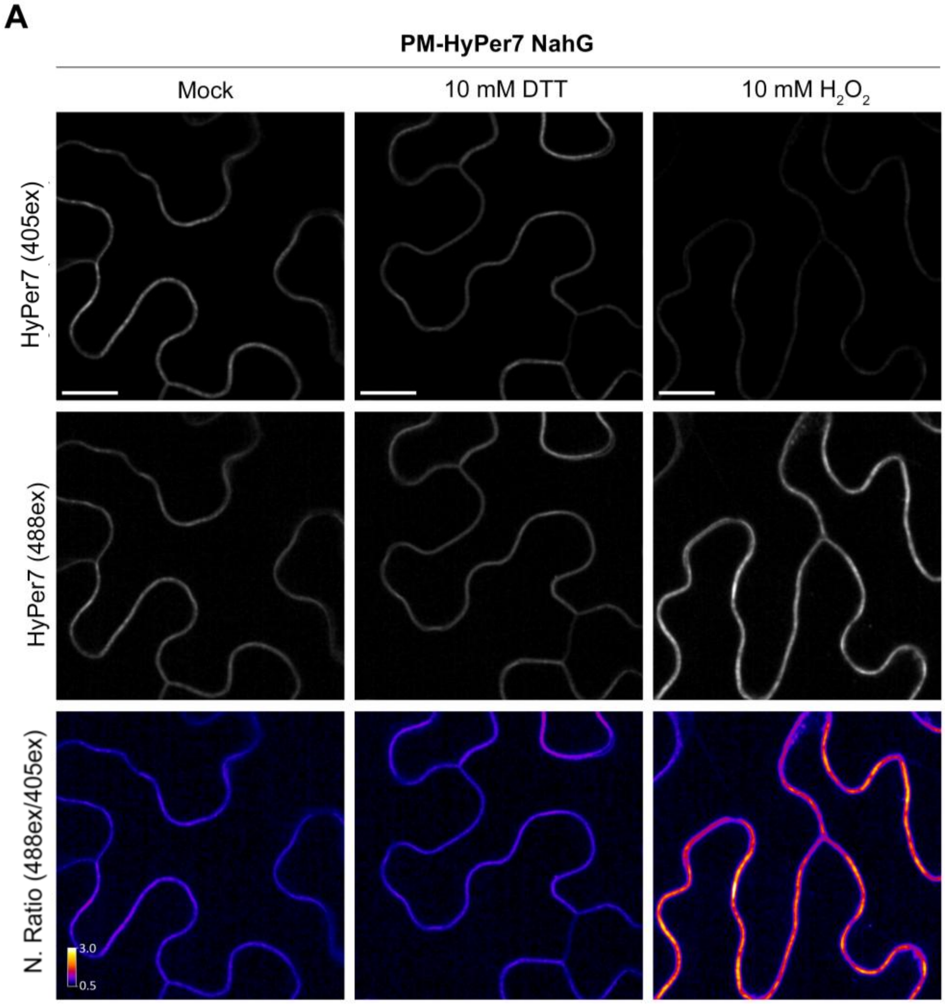

Supplementary figure 14, continued

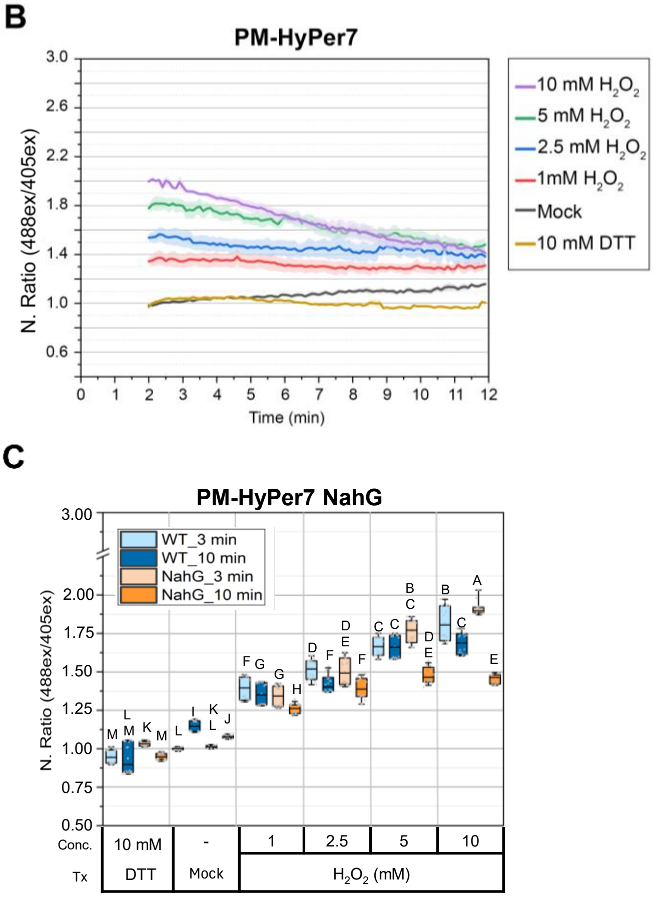

Time-lapse responses of PM-HyPer7 in *NahG* to various redox treatments. (A) Representative fluorescence images of PM-HyPer7 in *NahG*. Top panel, 405 nm excitation; middle panel, 488 nm excitation; and bottom panel, ratio image (488 nm ex./405 nm ex.). Images show responses to Mock, 10 mM DTT, and 10 mM H_2O_2 treatments at 3-4-mpt. Ratio values are represented by pseudocolor scale from 0.5 to 3.0. Scale bars, 20 µm. (B) Timelapse changes of fluorescence intensity ratios of PM-HyPer7 in *NahG* under different redox conditions. Data were collected a total of n = 6 images using at least four plants per treatment from two independent experiments. This experiment has been repeated three times and represented data from two experiments. (C) Box plot showing relative fluorescence ratios (488 nm ex./405 nm ex.) of PM-HyPer7 in WT and *NahG* leaf epidermal cells at early (3-4 min) and late (10-11 min) time points after each treatment. The ratios were normalized to those of mean ratios at 3-mpt in each experiment. Data was extracted from time-lapse data presented in B. Data were collected from a total of n = 6 images using at least four plants per treatment from two independent experiments. Statistical analysis was performed using Kruskal-Wallis tests followed by Conover's post-hoc tests with Benjamini-Hochberg correction for pairwise comparisons (α = 0.05). Different letters indicate statistically significant differences between groups ($p < 0.0001$). This figure supplements Figure 5 results.

Supplementary figure 15

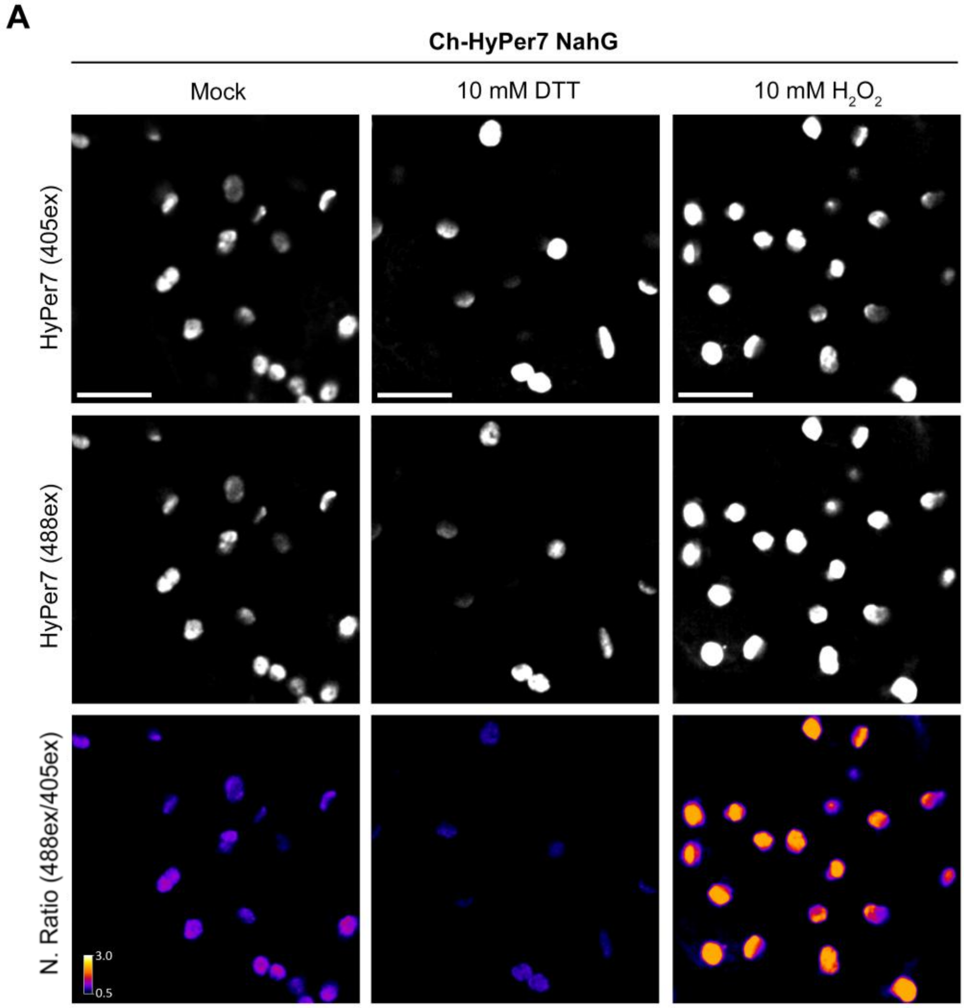

Supplementary figure 15, continued

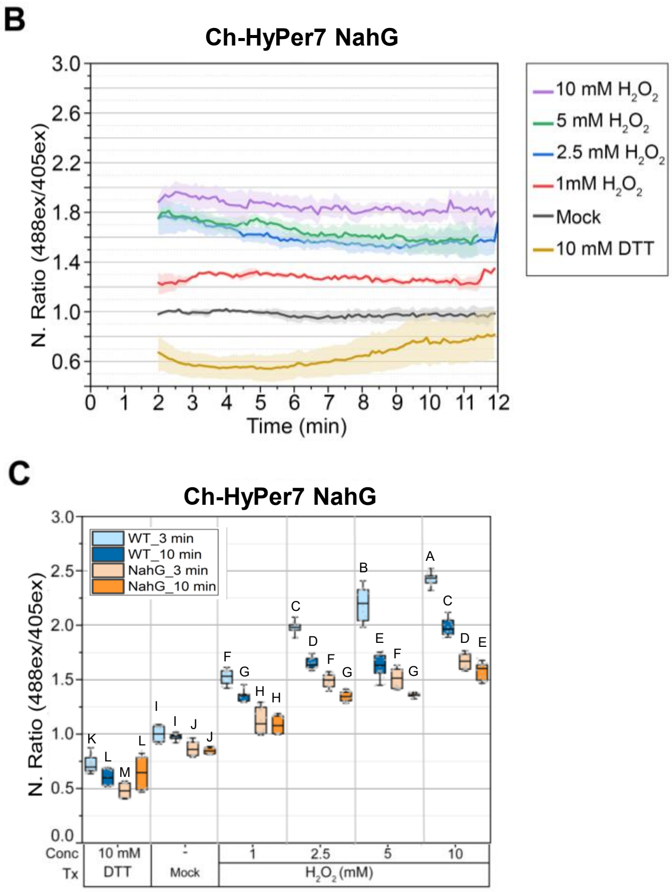

Time-lapse responses of Ch-HyPer7 in *NahG* to various redox treatments. (A) Representative fluorescence images of Ch-HyPer7 in *NahG*. Top panel, 405 nm excitation; middle panel, 488 nm excitation; and bottom panel, ratio image (488 nm ex./405 nm ex.). Images show responses to Mock, 10 mM DTT, and 10 mM H_2O_2 treatments at 3-4-mpt. Ratio values are represented by pseudocolor scale from 0.5 to 3.0. Scale bars, 20 µm. (B) Timelapse changes of fluorescence intensity ratios of Ch-HyPer7 in *NahG* under different redox conditions. Data were collected from a total of n = 6 images using at least four plants per treatment from two independent experiments. This experiment has been repeated three times and represented data from two replicate experiments. (C) Box plot showing relative fluorescence ratios (488 nm ex./405 nm ex.) of Ch-HyPer7 in WT and *NahG* leaf epidermal cells at early (3-4 min) and late (10-11 min) time points after each treatment. The ratios were normalized to those of mean ratios at 3-mpt in each experiment. Data was extracted from time-lapse data presented in B. Data was collected from a total of n = 6 images using at least four plants per treatment from two independent experiments. Statistical analysis was performed using Kruskal-Wallis tests followed by Conover's post-hoc tests with Benjamini-Hochberg correction for pairwise comparisons (α = 0.05). Different letters indicate statistically significant differences between groups ($p < 0.0001$). This figure supplements Figure 5 results.

Supplementary figure 16

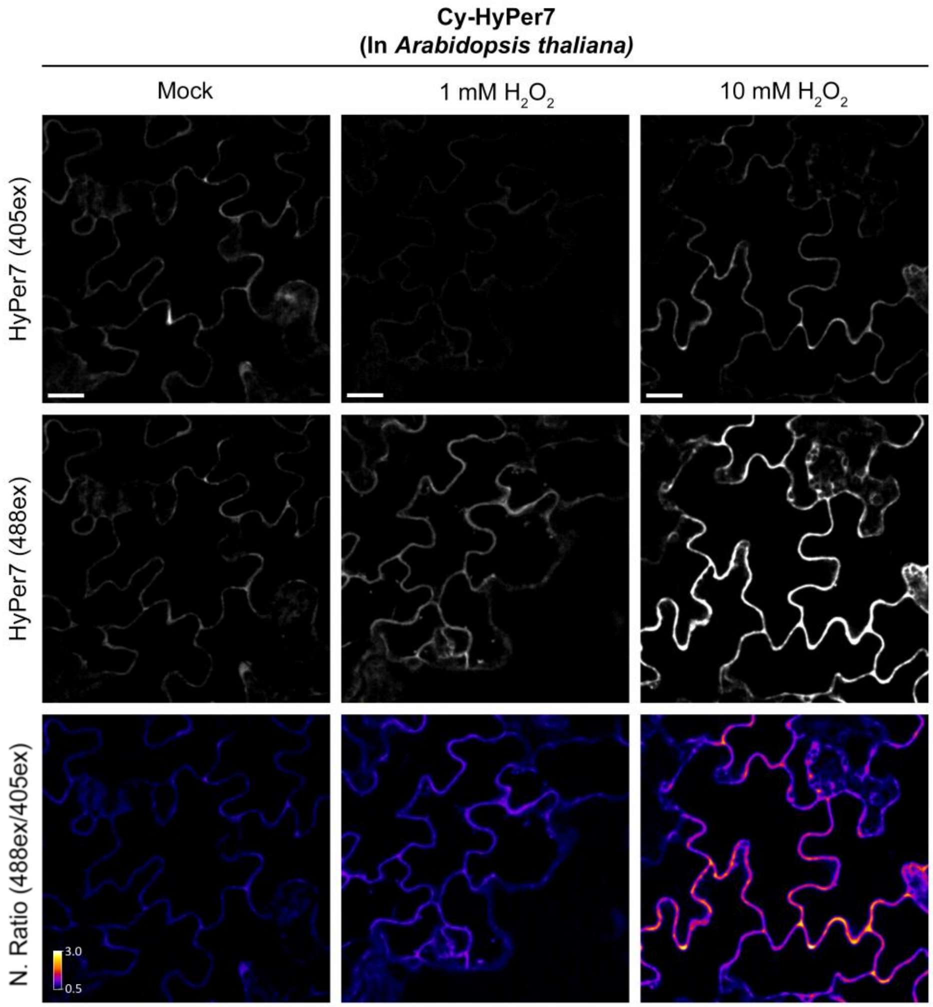

Responses of Cy-HyPer7 in *Arabidopsis* to H_2O_2. Representative fluorescence images of Cy-HyPer7 stably expressed in transgenic *Arabidopsis*. Flowering *Arabidopsis* Col-0 plants were transformed with the same binary vector used for transient expression in *N. benthamiana* and several transgenic lines were selected. T2 (line # 4) *Arabidopsis* seedlings vertically grown on ½ Murashige Skoog (MS) agar plates were used for treatments and ratio imaging at 7-days post imbibition. Images were taken on abaxial epidermal cells of cotyledons immediately after treatment. Top panel, 405 nm excitation; middle panel, 488 nm excitation; and bottom panel, ratio image (488 nm ex./405 nm ex.). Images show responses to Mock and 1- and 10-mM H_2O_2 treatments at 3-mpt. Ratio values are represented by pseudocolor scale from 0.5 to 3.0. Scale bars, 20 μm.

Supplementary figure 17

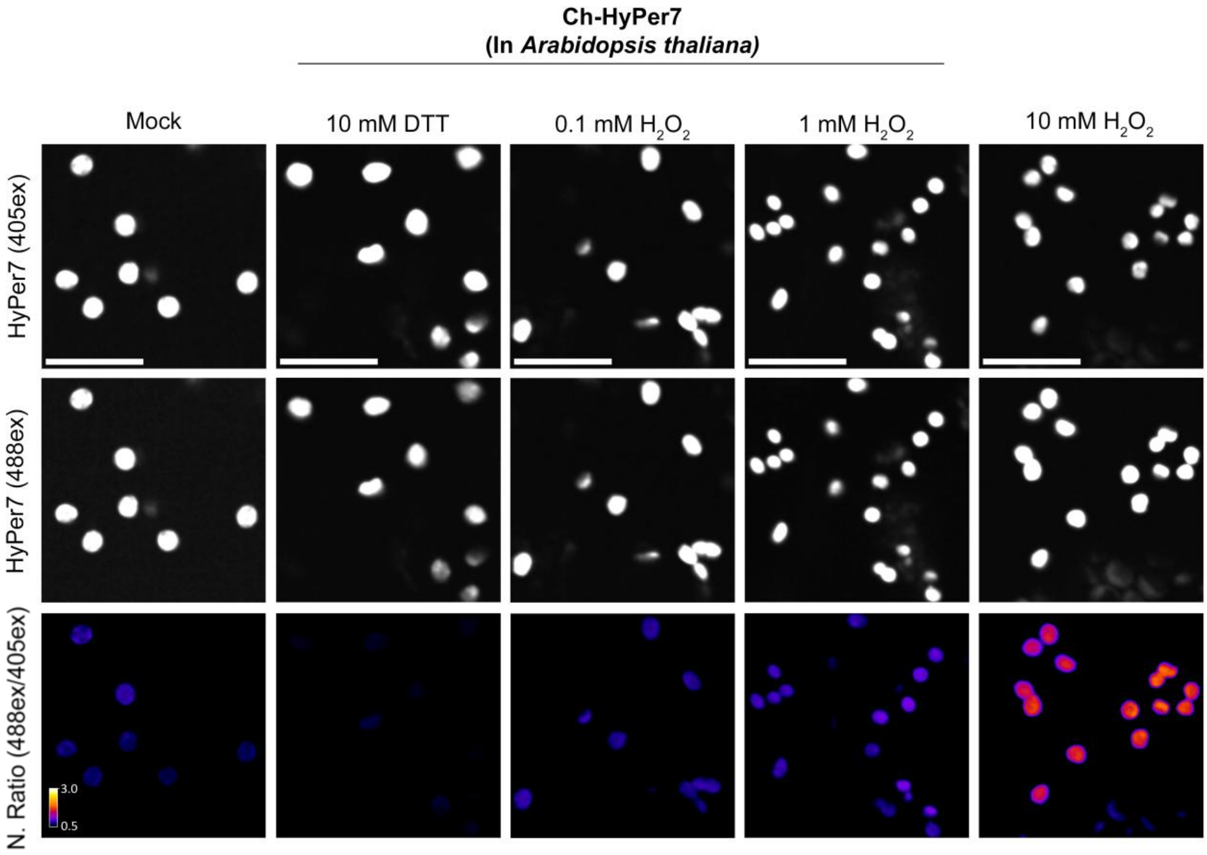

Responses of Ch-HyPer7 to redox treatments in *Arabidopsis.* Representative fluorescence images of Ch-HyPer7 stably expressed in transgenic *Arabidopsis*. Flowering *Arabidopsis* Col-0 plants were transformed with the same binary vector used for transient expression in *N. benthamiana* and several transgenic lines were selected. T2 (line # 3) *Arabidopsis* seedlings vertically grown on ½ Murashige Skoog (MS) agar plates were used for treatments and ratio imaging at 7-days post imbibition. Images were taken on abaxial epidermal cells of cotyledons immediately after treatment. Top panel, 405 nm excitation; middle panel, 488 nm excitation; and bottom panel, ratio image (488 nm ex./405 nm ex.). Images show responses to Mock (water), DTT, and H_2O_2 treatments at 3-mpt. Ratio values are represented by pseudocolor scale from 0.5 to 3.0. Scale bars, 20 μm.

Supplementary figure 18

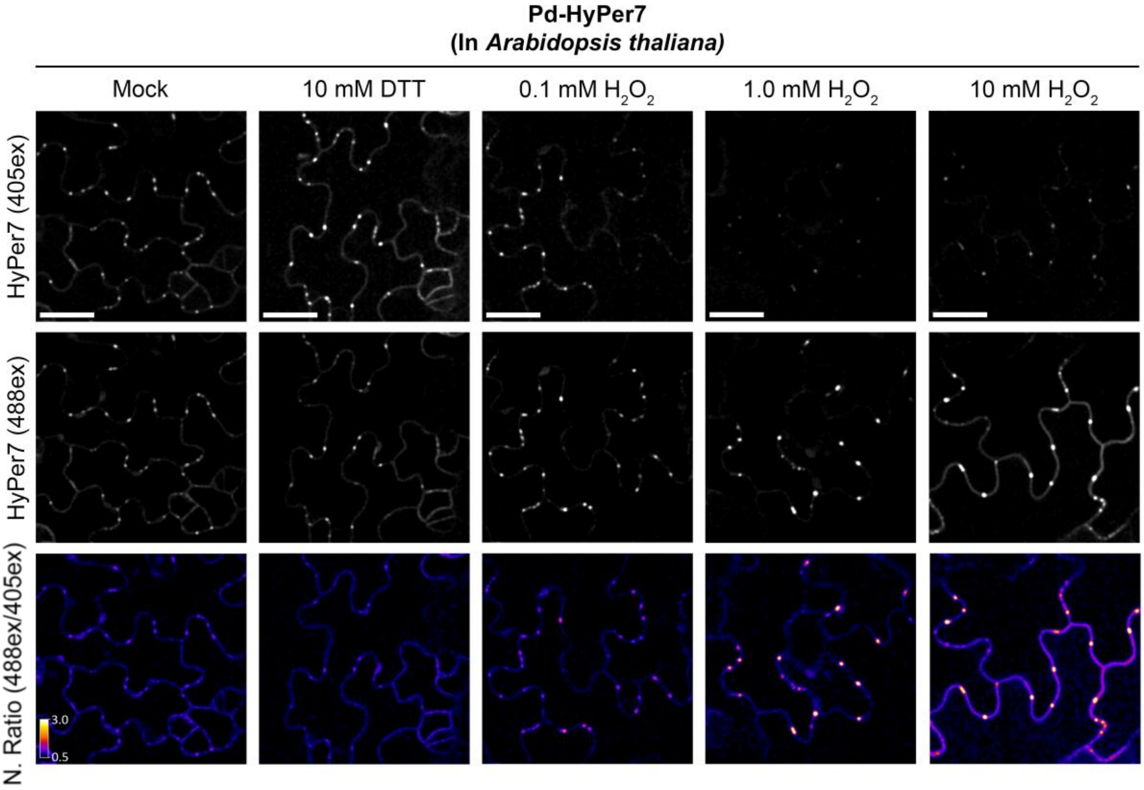

Responses of Pd-HyPer7 to redox treatments in *Arabidopsis.* Representative fluorescence images of Pd-HyPer7 stably expressed in transgenic *Arabidopsis*. Flowering *Arabidopsis* Col-0 plants were transformed with the same binary vector used for transient expression in *N. benthamiana* and several transgenic lines were selected. T2 or T3 (line # 27) *Arabidopsis* seedlings grown on ½ Murashige Skoog agar plates were used for treatments and ratio imaging at 7-days post imbibition. Images were taken on abaxial epidermal cells of cotyledons immediately after treatment. Top panel, 405 nm excitation; middle panel, 488 nm excitation; and bottom panel, ratio image (488 nm ex./405 nm ex.). Images show responses to Mock, DTT, and H_2O_2 treatments at 3-mpt. Ratio values are represented by pseudocolor scale from 0.5 to 3.0. Scale bars, 20 µm.

Supplementary figure 19

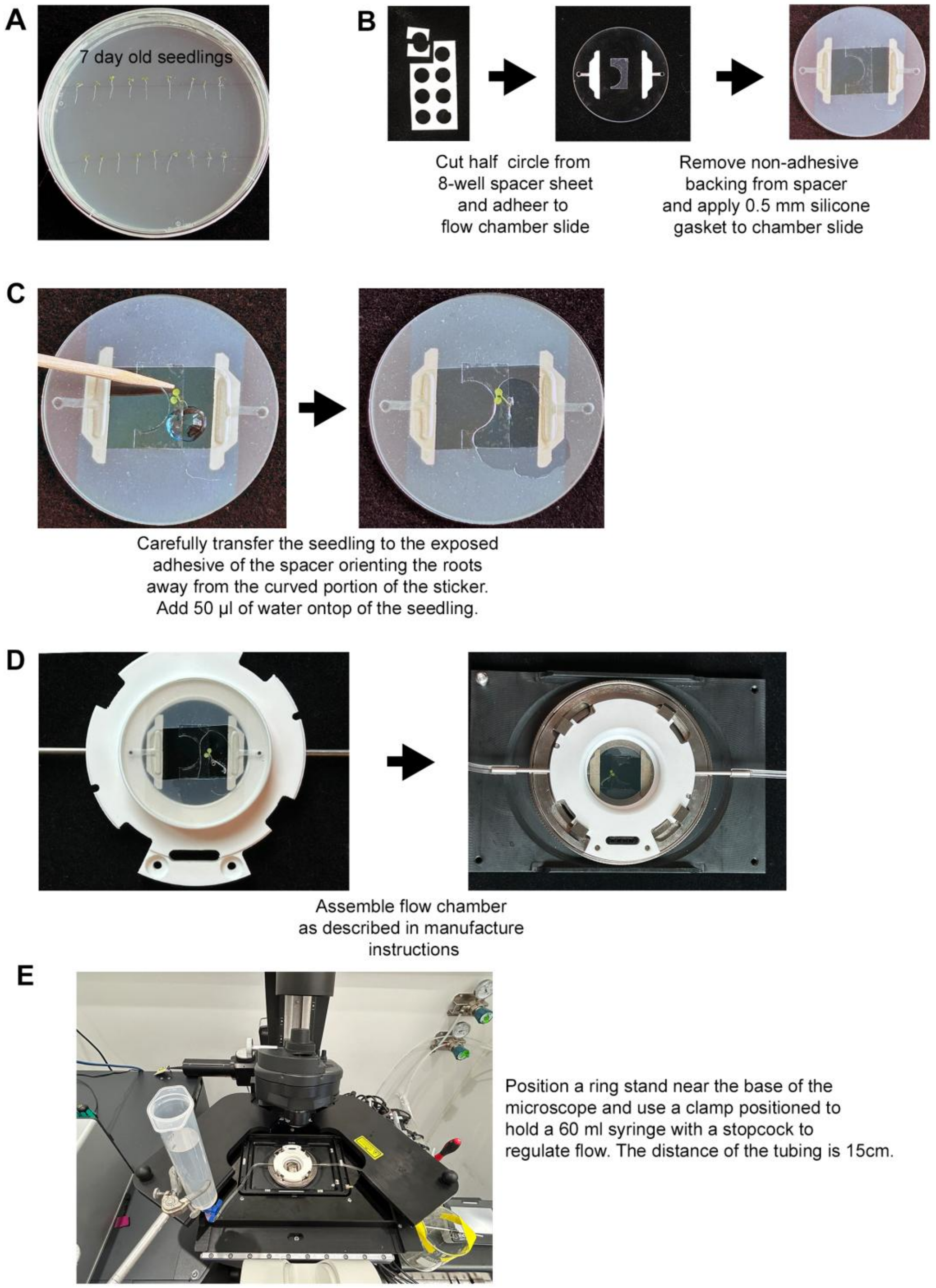

Construction and assembly of the flow chamber system.
